# Hnf4α integrates AIF and caspase 3/9 signaling to restrict single and coinfecting pathogens in teleosts

**DOI:** 10.1371/journal.ppat.1013491

**Published:** 2025-09-08

**Authors:** Dong Yan, Min Hui Tao, Xiao Man Wu, Jie Zhang, Ming Li, Ming Xian Chang

**Affiliations:** 1 State Key Laboratory of Breeding Biotechnology and Sustainable Aquaculture, Institute of Hydrobiology, Chinese Academy of Sciences, Wuhan, China; 2 College of Advanced Agricultural Sciences, University of Chinese Academy of Sciences, Beijing, China; University of Southern California, UNITED STATES OF AMERICA

## Abstract

Hepatocyte nuclear factor 4 alpha (Hnf4α), a conserved nuclear receptor central to vertebrate liver development and metabolic regulation, emerges here as a pivotal immune regulator in teleosts against complex infectious threats. While its metabolic roles are well-established, Hnf4α’s function in bacterial infection, viral infection, and bacterial-viral coinfection—major challenges in global aquaculture—remained uncharacterized. This study reveals that teleost Hnf4α acts as a dual-functional immune checkpoint, essential for combating *Aeromonas salmonicida*, grass carp reovirus (GCRV), and their coinfection. In *in vivo* zebrafish models, *hnf4α*-deficient larvae showed profound susceptibility, with survival rates reduced by 13.33–40% during infections, whereas gcHnf4α overexpression enhanced larval survival by 17.78–23.33% in single or coinfection scenarios. *In vitro* analyses in CIK cells demonstrated that gcHnf4α restricts *A. salmonicida* proliferation and GCRV replication through activation of a mitochondrial apoptotic program. Mechanistically, gcHnf4α forms a nuclear signaling complex with apoptosis-inducing factor (AIF) and caspases 3/9, driving a dual-dependent apoptotic pathway: (1) AIF-mediated caspase-independent nuclear apoptotic processes and (2) caspase 3/9-dependent cytoplasmic apoptotic execution. Confocal microscopy and co-immunoprecipitation validated direct interactions between gcHnf4α and these apoptotic effectors. Pharmacological inhibition of caspases 3/9 or AIF silencing abrogated gcHnf4α’s protective effects, while ectopic caspase expression rescued survival deficits in *hnf4α*-deficient larvae. These findings establish Hnf4α as a conserved molecular nexus linking nuclear receptor signaling to apoptotic immunity, offering a novel strategy for aquacultural disease control. By targeting the AIF-caspase axis, Hnf4α enables efficient pathogen elimination, delineating it as a promising target for developing dual-action immunomodulators.

## Author summary

As a key transcription factor for liver development, mammalian HNF4α is essential for the regulation of liver bile acid signaling and the progression of chronic liver disease. Although it is also well known that HNF4α promotes viral replication via binding to the HBV enhancers, little information is available about the role of vertebrate HNF4α in the regulation of host immune responses to control bacterial infection or bacterial-viral coinfections. This study reveals that early-stage bacterial, viral, or coinfection triggers rapid upregulation of *gchnf4α*, which in turn activates a conserved apoptotic cascade. Mechanistically, piscine Hnf4α directly interacts with the AIF-caspase 3/caspase 9 axis, driving dual-dependent apoptosis to restrict pathogen propagation. Functional validation demonstrates that Hnf4α overexpression enhances survival across infection types, while genetic ablation abolishes anti-pathogen defenses. Notably, we identified a teleost-specific adaptation where Hnf4α’s DNA-binding domain (DBD) engages AIF for caspase-independent nuclear apoptotic event, whereas its ligand-binding domain (LBD) activates caspase 3/9 for canonical apoptosis. These findings expand the canonical functions of vertebrate HNF4α beyond metabolism, establishing it as a critical immune regulator. The discovery of the AIF-caspase axis as a central mediator of Hnf4α’s protective effects provide a mechanistic basis for developing targeted immunotherapies. Given the global burden of infectious diseases in aquaculture, Hnf4α represents a promising therapeutic target for enhancing host resilience against complex pathogen challenges.

## Introduction

Hepatocyte nuclear factor 4-alpha (HNF4α), first isolated from mammalian liver nuclei in the 1990s, is a prototypical nuclear receptor (NR) and master regulator of liver-specific gene expression [1,2]. As a member of the NR superfamily, HNF4α exhibits evolutionary conservation across vertebrates, characterized by a DNA-binding domain (DBD) and a ligand-binding domain (LBD) at the C-terminus [3]. In mammals, its genomic locus contains two distal promoters (P1 and P2, separated by >45 kb), driving tissue-specific alternative splicing to generate 12 isoforms classified into P1 (α1–α6) and P2 (α7–α12) subgroups. These isoforms mediate spatiotemporal gene regulation in liver, intestine, and kidney, underscoring HNF4α’s role in developmental and metabolic homeostasis [4–6].

As a pivotal regulator of hepatic development and metabolism, mammalian HNF4α is intricately linked to liver pathobiology. Genetic and clinical studies have established its involvement in hepatitis, cirrhosis, and hepatocellular carcinoma (HCC) [7,8]. HNF4α modulates inflammatory and metabolic pathways by regulating genes involved in lipid/cholesterol metabolism and drug detoxification, directly influencing the liver’s response to viral or toxic stresses [9,10]. Dysregulated HNF4α expression accelerates hepatic fibrosis by impairing hepatocyte proliferation and promoting fibrogenic signaling, contributing to the loss of metabolic function and tissue architecture [11,12]. Aberrant HNF4α isoform expression correlates with malignant transformation, modulating oncogenic pathways to influence hepatocellular proliferation, invasion, and metastasis [13,14].

In teleost fish, Hnf4α research has primarily focused on metabolic regulation. For example, rabbitfish (*Siganus canaliculatus*) Hnf4α upregulates Δ4 Fad, Δ4 fads2, and elovl5 to drive long-chain polyunsaturated fatty acid (LC-PUFA) biosynthesis [15–19], while gilthead sea bream (*Sparus aurata*) Hnf4α transactivates the mitochondrial alanine aminotransferase (ALT) gene critical for amino acid metabolism [20]. In zebrafish, Hnf4α maintains intestinal homeostasis by activating microbial growth-inhibitory transcriptional enhancers [21]. Despite these insights, the role of piscine Hnf4α in bacterial infection and bacterial-viral coinfection—common challenges in aquaculture—remains uncharacterized.

Grass carp (*Ctenopharyngodon idella*), a staple of China’s freshwater aquaculture, plays a pivotal role in ensuring aquatic product security and driving fisheries development; however, its high susceptibility to pathogens—particularly grass carp reovirus (GCRV) and bacterial pathogens—frequently causes outbreaks, incurring substantial economic losses. GCRV, the etiological agent of grass carp viral hemorrhagic disease, belongs to the *Reoviridae* family, with three genotypes (GCRV-I, -II, -III) distinguished by marked divergence in core structural genes, alongside striking differences in virulence, cell tropism, and pathogenicity [22,23]. GCRV-I and -III are low-virulence strains, rarely inducing clinical disease *in vivo* but exhibiting robust cytopathic effects (CPE) in grass carp kidney (CIK) cells—making them tractable for *in vitro* studies. In contrast, GCRV-II is a high-virulence genotype characterized by a short latent period and lethal outbreaks in juvenile grass carp, yet it lacks a sensitive cell line model, limiting mechanistic investigations of its pathogenicity [22–24]. This disparity necessitated our experimental design: GCRV-I for dissecting conserved viral replication processes *in vitro*, and GCRV-II for modeling acute *in vivo* pathogenesis. Beyond single-pathogen infections, polymicrobial disease poses an escalating threat to grass carp aquaculture. Viral hemorrhagic disease frequently co-occurs with bacterial pathogens such as *Aeromonas hydrophila*, driving large-scale, persistent mortality [25,26]. Although vaccines or traditional Chinese medicines have achieved certain success in preventing infections caused by a single pathogen, the effective prevention and control methods for co-infection with GCRV - *Aeromonas* are quite limited.

*Aeromonas salmonicida* was isolated from moribund grass carp with severe pathological symptoms and preserved by our laboratory [27]. The nuclear receptor Hnf4 family comprises three isoforms—Hnf4α, Hnf4β, and Hnf4γ—with Hnf4β exhibiting a restricted phylogenetic distribution, being conserved in teleosts, amphibians, and avian species but absent in mammals [28]. Recent findings have implicated Hnf4β as a key mediator of antibacterial immunity in teleosts, demonstrating that grass carp Hnf4β (gcHnf4β) restricts *A. salmonicida* infection through direct interaction with apoptosis-inducing factor (AIF) and formation of a ternary complex with grass carp Hnf4α (gcHnf4α) to indirectly recruit caspase 3 [27]. Building on this foundation, the present study interrogates the distinct and collaborative roles of gcHnf4α in (i) modulating host defenses against *A. salmonicida* mono-infection and (ii) shaping the immunopathogenesis of co-infection with GCRV. By dissecting the dual functionality of gcHnf4α in these contexts, we aim to uncover novel molecular checkpoints governing teleost immunity and identify potential targets for intervention against polymicrobial diseases in fish farming. Here, we demonstrate that gcHnf4α forms a functional complex with AIF and caspases 3/9, enhancing caspase activity in an AIF-dependent manner to promote apoptosis. Pharmacological inhibition of caspases 3/9 abrogates gcHnf4α-mediated antibacterial and antiviral responses, while overexpression of caspases 3/9 rescues survival defects in *zfhnf4α*-deficient zebrafish larvae during bacterial challenge or coinfection. These findings establish a conserved apoptotic signaling axis through which piscine Hnf4α coordinates host defense against bacterial and mixed infections, bridging gaps in teleost immune regulation and expanding the functional repertoire of this ancient nuclear receptor.

## Results

### Sequence characteristics, expression and subcellular localization analysis of gcHnf4α

To functionally characterize gcHnf4α (GenBank accession number: MH751509), gene-specific primers were designed to amplify its full open reading frame (ORF) from grass carp cDNA. Amplification using the first ATG in the *hnf4α*-containing ORF failed, but targeting the second ATG yielded a 1,362-bp product matching the database sequence, encoding a 454-amino acid polypeptide (predicted MW: 49.9 kDa). Structural analysis confirmed canonical nuclear receptor architecture, including a conserved N-terminal DBD (residues 51–126) and a C-terminal LBD (residues 142–363) containing hallmark α-helical motifs.

Zebrafish Hnf4α (zfHnf4α) and mammalian Hnf4α exhibit multiple splicing isoforms. zfHnf4α isoforms range in length from 427–468 amino acids (predicted MW: 46.97–51.48 kDa; S1 Appendix). Cloned gcHnf4α shares 90.43%–97.58% sequence similarity with zfHnf4α and its isoforms, with 93.83 ~ 100% identity in the DBD and 97.75% identity in the LBD (S1 Table). In mammals, all splicing isoforms of human Hnf4α or mouse Hnf4α retain identical amino acid sequences in both the DBD and LBD. In contrast, teleost Hnf4α orthologs and their isoforms exhibit distinct structural divergence in these functional domains, as revealed by multiple sequence alignments of gcHnf4α, zfHnf4α and its splicing isoforms against mammalian counterparts (S1 Fig). Within the DBD: Zebrafish isoforms (zfHnf4α_tv1, tv3, tv4, tv6) display five amino acid insertions and three substitutions relative to mammalian DBDs, whereas gcHnf4α, zfHnf4α and its other isoforms (zfHnf4α_tv2, tv5, tv7) shows only two substitutions compared to mammals (S1A Fig). In the LBD: All fish Hnf4α orthologs (including gcHnf4α and zfHnf4α isoforms) share a conserved pattern of divergence from mammals, characterized by one amino acid deletion and 28 substitutions (S1B Fig). These lineage-specific modifications in DBD and LBD sequences—more pronounced in zebrafish isoforms than in gcHnf4α—highlight differential structural refinement of Hnf4α across teleosts, potentially underlying functional specialization in antimicrobial immunity distinct from mammalian. Phylogenetic analysis clustered teleost Hnf4α into a distinct clade, separate from mammalian orthologs, indicating functional divergence (S2A Fig).

A polyclonal antibody was generated against a conserved C-terminal polypeptide shared by gcHnf4α and all zfHnf4α isoforms (S1 Appendix), enabling cross-recognition: it detected a ~ 50 kDa band corresponding to gcHnf4α-FLAG in grass carp CIK cells (S2B Fig). In wild type zebrafish cells from the tail fin, anti-Hnf4α antibody could detect a ~ 50 kDa band and a slightly larger band corresponding to zfHnf4α-FLAG (GenBank accession number: NP_919349).

**Fig 1:**
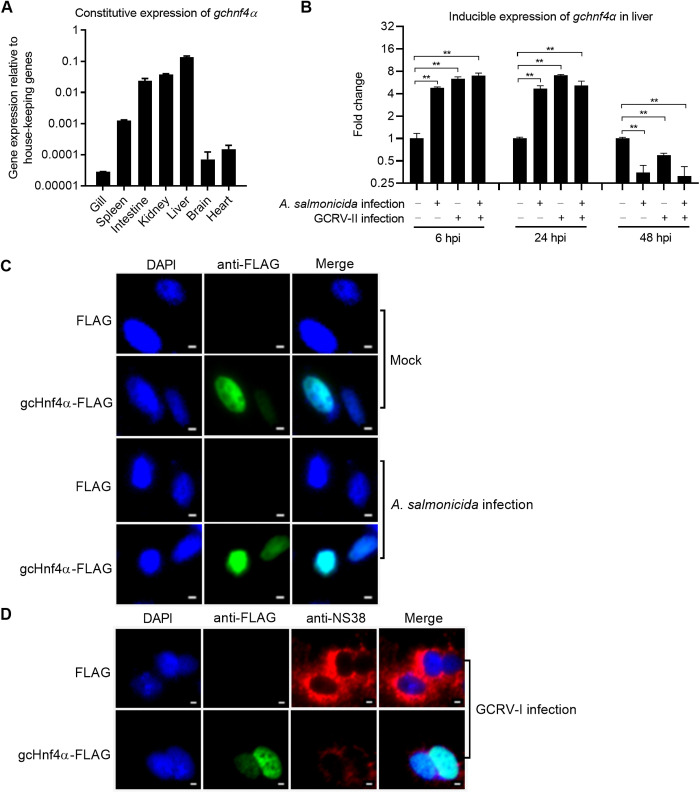
Expression and subcellular localization of grass carp Hnf4α. (A) Constitutive expression of *gchnf4α* in grass carp tissues. Gill, spleen, intestine, kidney, liver, brain, and heart from three healthy fish were analyzed by qRT-PCR. (B) Inducible expression of *gchnf4α* in the liver following infection with *A. salmonicida*, GCRV-II, or coinfection. Liver samples were collected at 6, 24, and 48 hpi from three fish per group and analyzed by qRT-PCR. Data are presented as mean ± SEM (n = 3). (C-D) Subcellular localization of gcHnf4α in CIK cells with or without *A. salmonicida* (C) or GCRV-I (D) infection (MOI = 0.5). Cells were visualized by confocal microscopy. Scale bars = 2 μM. Statistical significance was determined by Student’s t-test (** *p* < 0.01).

To analyze the tissue-specific expression of *gchnf4α*, quantitative real-time PCR (qRT-PCR) was performed on seven grass carp tissues: gill, spleen, intestine, kidney, liver, brain, and heart. As shown in Fig 1A, *gchnf4α* mRNA exhibited the highest abundance in the liver, followed by the kidney and intestine, with the lowest levels detected in the gill.

Next, we assessed *gchnf4α* transcriptional responses to monoinfection (with *A. salmonicida* or GCRV-II) and coinfection (with both pathogens simultaneously). Liver samples collected at 6, 24, and 48 hours post-infection (hpi) were analyzed by qRT-PCR. At early time points (6 and 24 hpi), *gchnf4α* mRNA was significantly upregulated in all infected groups compared to uninfected controls. Conversely, at 48 hpi, *gchnf4α* expression was downregulated in *A. salmonicida*-infected, GCRV-II-infected, and coinfected fish (Fig 1B).

Subcellular localization of gcHnf4α was examined in CIK cells via confocal immunofluorescence. Given that GCRV-I (but not GCRV-II) induces robust cytopathic effects in CIK cells, GCRV-I was used for infection studies [24]. gcHnf4α exhibited exclusive nuclear localization, with no detectable shift in subcellular distribution following *A. salmonicida* or GCRV-I infection (Fig 1C and 1D). Viral inclusion bodies (VIBs), critical for GCRV RNA synthesis, rely on the nonstructural protein NS38 for assembly in both GCRV-I and GCRV-II infections [22,29,30]. Notably, the obvious reduction in NS38 immunofluorescence intensity (Fig 1D) suggested that gcHnf4α suppressed VIBs formation, a finding corroborated by supplemental data (S3 Fig). Quantification of VIBs in CIK cells overexpressing gcHnf4α revealed a significant decrease compared to control cells (S3C and S3D Fig), indicating gcHnf4α’s inhibitory role in VIBs biogenesis. These findings suggest that nuclear-localized gcHnf4α may interfere with pathogen proliferation.

### gcHnf4α exhibits protective roles in antibacterial and antiviral responses during *A. salmonicida* infection, GCRV-I infection, and coinfection

To characterize the *in vitro* functional role of gcHnf4α in host defense against bacterial, viral, and coinfectious challenges, CIK cells were transfected with either empty FLAG vector or gcHnf4α-FLAG plasmid, followed by infection with *A. salmonicida*, GCRV-I, or both pathogens simultaneously.

**Fig 2:**
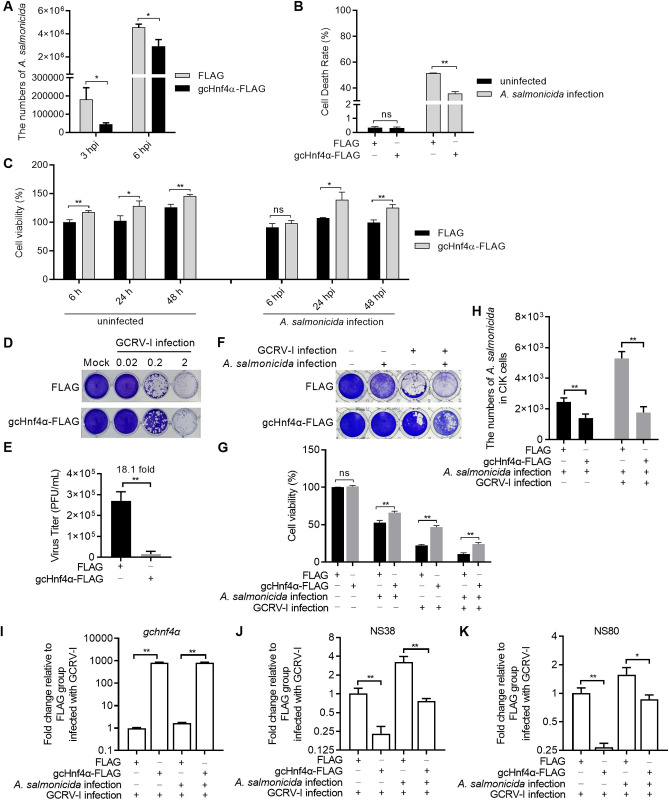
Grass carp Hnf4α mediates antibacterial and antiviral effects *in vitro.* (A) gcHnf4α overexpression inhibits *A. salmonicida* proliferation in CIK cells. Cells were transfected with gcHnf4α-FLAG or vector control, infected at MOI = 0.5, and CFU enumerated at 3 and 6 hpi. (B) Reduced LDH release in gcHnf4α-overexpressing cells infected with *A. salmonicida* (MOI = 0.5, 6 hpi). (C) Enhanced cell viability in gcHnf4α-overexpressing cells during *A. salmonicida* infection, measured by CCK-8 at indicated time points. (D-E) gcHnf4α overexpression reduces GCRV-I cytopathic effect (MOI = 0.02–2, 18 hpi) and viral titers (MOI = 0.2). (F-G) Improved cell survival and viability in gcHnf4α-overexpressing cells during single or coinfection. (H) Reduced bacterial proliferation in gcHnf4α-overexpressing cells during single or coinfection. (I-K) Reduced viral gene (NS38, NS80) expression in gcHnf4α-overexpressing cells at 24 hpi. For all panels, data are mean ± SEM (n = 3). Statistical significance was determined by Student’s t-test (*p < 0.05, **p < 0.01).

During *A. salmonicida* mono - infection, compared to CIK cells transfected with the FLAG empty plasmid, overexpression of gcHnf4α significantly inhibited the proliferation of *A. salmonicida* (Fig 2A). Concomitantly, the cell death rate decreased, and the cell survival rate increased in gcHnf4α - overexpressing CIK cells (Fig 2B and 2C). In the case of GCRV-I mono - infection, compared to cells transfected with the FLAG empty plasmid, gcHnf4α overexpression enhanced the cells’ resistance to viral infection, as evidenced by a lower viral titer (Fig 2D and 2E).

During *A. salmonicida* – GCRV-I coinfection, when compared to CIK cells transfected with the FLAG empty plasmid, overexpression of gcHnf4α led to increased cell survival and decreased *A. salmonicida* proliferation (Fig 2F-2H). Bacterial pathogens can compromise virus particle stability through the release of proteolytic enzymes, reactive oxygen species, or other metabolites. Additionally, bacterial-induced cytolysis, necrosis, or apoptosis may overlap with viral CPE, rendering it impossible to distinguish virus-mediated cellular lesions from bacterial cytotoxicity in coinfected cells. This ambiguity leads to unreliable plaque counting and inaccurate determination of CPE endpoints in traditional virological assays. In contrast, the non-structural proteins NS38 and NS80—critical components of cytoplasmic GCRV VIBs—serve as robust markers of active viral replication [22]. Their expression is tightly coupled to viral transcription and the biogenesis of replication-competent VIBs. By targeting NS38 and NS80 transcripts via qRT-PCR, we exploit the genetic uniqueness of these viral genes—absent from both host and bacterial genomes—to avoid cross-reactivity with microbial or cellular nucleic acids. qRT-PCR analysis revealed that gcHnf4α overexpression suppressed the expression of NS38 and NS80 (Fig 2I-2K).

Collectively, these results suggest that gcHnf4α plays a comparable role in antibacterial and antiviral responses during *A. salmonicida* infection, GCRV-I infection, and *A. salmonicida* – GCRV-I coinfection.

### gcHnf4α induces apoptosis-associated gene expression and caspase 3/9 activation during *A. salmonicida* infection, GCRV-I infection, and coinfection

**Fig 3:**
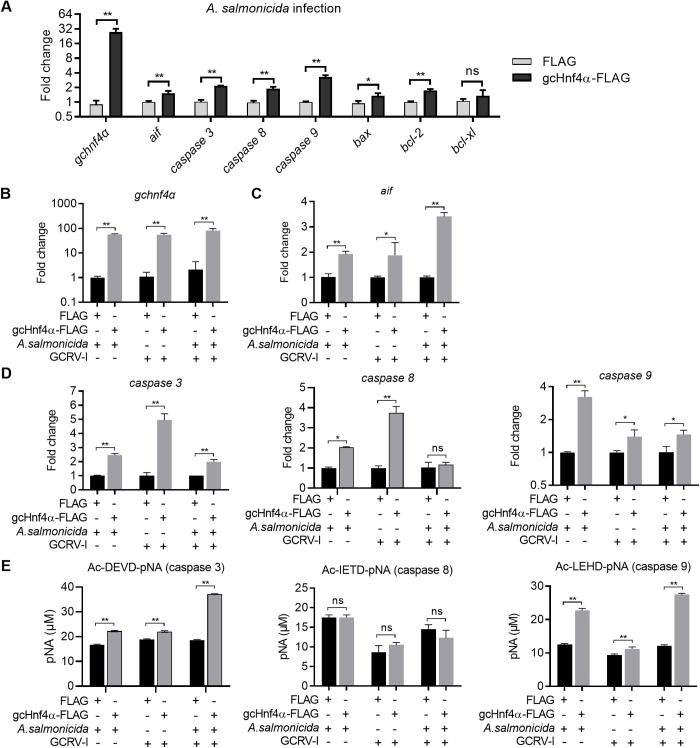
Grass carp Hnf4α induces the expression and activities of caspase 3 and caspase 9 *in vitro.* (A) gcHnf4α overexpression upregulates the expression levels of apoptosis-related genes during *A. salmonicida* infection. CIK cells were transfected with gcHnf4α-FLAG or empty vector for 36 h, infected at MOI = 0.5, and harvested at 6 hpi for qRT-PCR analysis. (B-D) gcHnf4α overexpression upregulates the expression levels of *aif*, *caspase 3* and *caspase* 9 both in single (*A. salmonicida* or GCRV-I) and coinfection models. CIK cells were transfected for 36 h, infected with pathogens at MOI = 0.5, and collected at 24 hpi for qRT-PCR. (E) gcHnf4α overexpression enhances enzymatic activity of caspase 3 and 9 in single (*A. salmonicida* or GCRV-I) and coinfection models. CIK cells were transfected for 36 h, infected with pathogens at MOI = 0.5, and collected at 24 hpi for caspase activity assays. Caspase 3/8/9 activities were evaluated using chromogenic substrates including Ac-DEVD-pNA (caspase 3), Ac-IETD-pNA (caspase 8), or Ac-LEHD-pNA (caspase 9). Data are mean ± SEM (n = 3). Statistical significance was determined by Student’s t-test (*p < 0.05, **p < 0.01; ns = not significant).

In mammals, HNF4α regulates a diverse transcriptome encompassing immune response, apoptosis, and stress adaptation pathways [31]. Given that both *A. salmonicida* and GCRV infections induce host cell apoptosis to facilitate pathogenesis [32–34], we investigated whether gcHnf4α modulates apoptotic signaling to restrict microbial invasion. qRT-PCR analysis revealed that gcHnf4α overexpression upregulated the mRNA levels of apoptosis-related genes—*aif, caspase 3*, c*aspase 8*, *caspase 9*, *bax*, and *bcl-2*—during *A. salmonicida* mono - infection (Fig 3A), indicating potential engagement of both pro- and anti-apoptotic pathways.

Following the initial quantification of apoptotic gene expression in bacterial infections, subsequent analyses focused on *aif* and *caspase* 3/8/9, as these molecules represent functionally distinct yet interconnected nodes of the apoptotic machinery: AIF mediates caspase-independent intrinsic apoptosis, while caspase 3 (effector), caspase 8 (extrinsic pathway initiator), and caspase 9 (intrinsic pathway initiator) collectively orchestrate caspase-dependent apoptotic signaling. During mono - infection (with *A. salmonicida* or GCRV-I) and coinfection, gcHnf4α overexpression consistently increased *aif, caspase 3* and *caspase 9* mRNA levels, with *caspase 8* expression induced only during single-pathogen infections (Fig 3B-3D).

To dissect functional consequences, we assayed caspase enzymatic activities using substrate-specific peptides: Ac-DEVD-pNA for caspase 3, Ac-IEVD-pNA for caspase 8, and Ac-LEVD-pNA for caspase 9. Enzymatic activity assays mirrored these trends: caspase 3/9 activities were significantly elevated in all infection models, whereas caspase 8 activity remained unaltered (Fig 3E).

Collectively, these findings demonstrate that gcHnf4α promotes apoptosis during microbial challenges by transcriptionally upregulating key apoptotic genes and functionally activating caspase 3/9, establishing a mechanistic link between nuclear receptor signaling and the execution phase of apoptosis of relevance to antibacterial and antiviral immunity.

### gcHnf4α interacts with caspase 3 and caspase 9 via its LBD to inhibit pathogen proliferation

**Fig 4:**
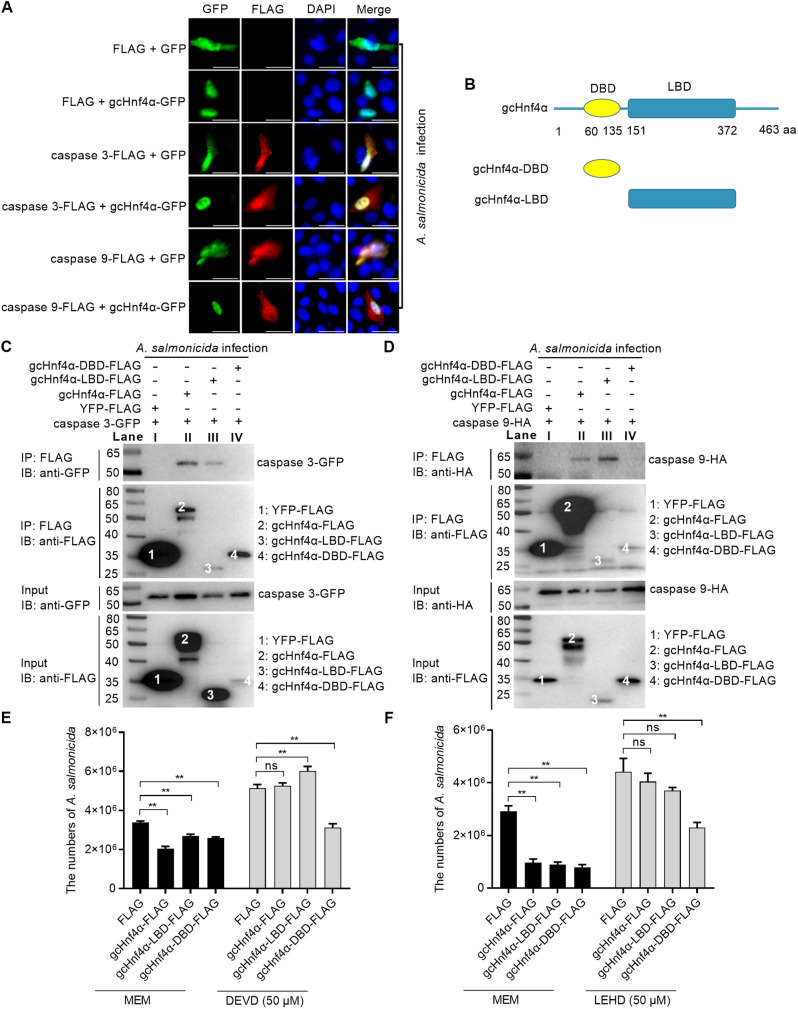
Grass carp Hnf4α associates with caspase 3 and caspase 9 for inhibiting pathogen proliferation via its ligand binding domain (LBD). (A) Subcellular colocalization of gcHnf4α with caspase 3/9 in *A. salmonicida*-infected CIK cells (MOI = 0.5, 6 hpi). Scale bar = 20 μM. (B) Schematic diagrams of gcHnf4α and its mutants including gcHnf4α-DBD and gcHnf4α-LBD. (C-D) Co-immunoprecipitation confirms interaction between gcHnf4α (full-length/LBD/DBD) and caspase 3/9. (E-F) Caspase 3/9 inhibition abrogates gcHnf4α-mediated *A. salmonicida* growth restriction (MOI = 0.5, 6 hpi). Data are mean ± SEM (n = 3). Statistical significance was determined by Student’s t-test (*p < 0.05, **p < 0.01; ns = not significant).

Given gcHnf4α’s conserved protective role and its functional association with caspase 3/9 across *A. salmonicida*, GCRV-I, and coinfection models (Figs 2 and 3), we focused on dissecting their molecular interaction during bacterial infection. Caspase 3 localizes primarily in the cytoplasm but translocates to the nucleus upon activation [35], while caspase 9 exhibits punctate cytoplasmic and nuclear staining [36]. Confocal immunofluorescence revealed that gcHnf4α—predominantly nuclear—exhibits robust nuclear colocalization with both caspases (Fig 4A), consistent with their potential functional interplay.

To map the interacting domain, we generated FLAG-tagged gcHnf4α truncation constructs encoding either the DBD or LBD (Fig 4B). Co-immunoprecipitation (Co-IP) assays validated direct physical interactions: CIK cells co-transfected with gcHnf4α-FLAG and caspase 3-GFP or caspase 9-HA were lysed, and immunoprecipitation with anti-FLAG beads pulled down the FLAG-tagged gcHnf4α along with co-expressed caspase 3-GFP (detected by anti-GFP antibody, Fig 4C, Lane II) and caspase 9-HA (detected by anti-HA antibody, Fig 4D, Lane II). Notably, only the LBD-FLAG construct, not DBD-FLAG, associated with caspase 3-GFP (detected by anti-GFP antibody, Fig 4C, Lane III) and caspase 9-HA (detected by anti-HA antibody, Fig 4D, Lane III), identifying the LBD as the critical interaction domain.

Subsequently, we investigated the antibacterial mechanism of gcHnf4α. Analogous to the full-length gcHnf4α, truncated constructs of gcHnf4α expressing either the DBD or the LBD also displayed antibacterial capabilities. To assess the role of caspases in this antibacterial process, we used specific inhibitors. When we employed DEVD to inhibit caspase 3 activity, the antibacterial function of both the full-length gcHnf4α and the truncated construct expressing the LBD was completely abolished. In contrast, the antibacterial ability of the truncated construct expressing the DBD remained unaffected (Fig 4E). Similarly, when we treated the cells with LEHD to inhibit caspase 9 activity, the antibacterial capacity of the full-length gcHnf4α and the LBD-expressing truncated construct was severely hampered. However, the DBD expressing truncated construct’s antibacterial function remained intact (Fig 4F).

Given the availability of only Hnf4α and caspase 9 antibodies validated for grass carp, we focused on investigating their endogenous interaction in CIK cells. In input lysates from uninfected, *A. salmonicida*/GCRV-II single-infected, or co-infected cells, the anti-caspase 9 antibody detected full-length caspase 9 (46 kDa) and a weakly expressed cleaved form (~37 kDa). An Hnf4α antibody, raised against a sequence conserved between zebrafish and grass carp Hnf4α, could recognize endogenous gcHnf4α in CIK cells (S2B Fig). Co-IP with this Hnf4α antibody-conjugated beads revealed robust binding to full-length caspase 9, with faint interaction with the cleaved form observable upon prolonged exposure (S4A Fig). Importantly, the antibody-bead complex did not precipitate the unrelated housekeeping protein GAPDH (S4A Fig), confirming the specificity of the gcHnf4α-caspase 9 interaction. Notably, this endogenous gcHnf4α-caspase 9 interaction remained consistent across all experimental conditions—unaffected by pathogen type (*A. salmonicida* or GCRV-II), infection mode (single or co-infection), or pathogenic stimulation itself—indicating a constitutive rather than infection-inducible association that likely functions as a preassembled signaling hub to enable rapid apoptotic activation during microbial challenge.

To determine whether gcHnf4α-mediated antiviral activity depends on caspase 3 and caspase 9, we evaluated the role of these caspases in gcHnf4α-driven viral restriction. In DMSO-treated cells, full-length gcHnf4α overexpression significantly reduced GCRV load, whereas expression of its individual domains (DBD or LBD alone) had no effect—indicating that, unlike its antibacterial activity (which is mediated by individual domains), gcHnf4α’s antiviral function requires both intact DBD and LBD. Notably, in cells treated with the caspase 3 inhibitor DEVD, gcHnf4α overexpression instead promoted viral replication, with no significant effects from its isolated domains. Similarly, caspase 9 inhibition (via LEHD) abrogated gcHnf4α’s antiviral activity, as neither full-length gcHnf4α nor its domains affected viral load (S4B Fig).

Collectively, these findings indicate that gcHnf4α relies on the activities of caspase 3 and caspase 9 to inhibit pathogen proliferation. This inhibitory effect is mediated by the interaction between the LBD of gcHnf4α and caspase 3/9.

### gcHnf4α-dependent activation of caspase 3/9 via AIF mediates pathogen proliferation inhibition

**Fig 5:**
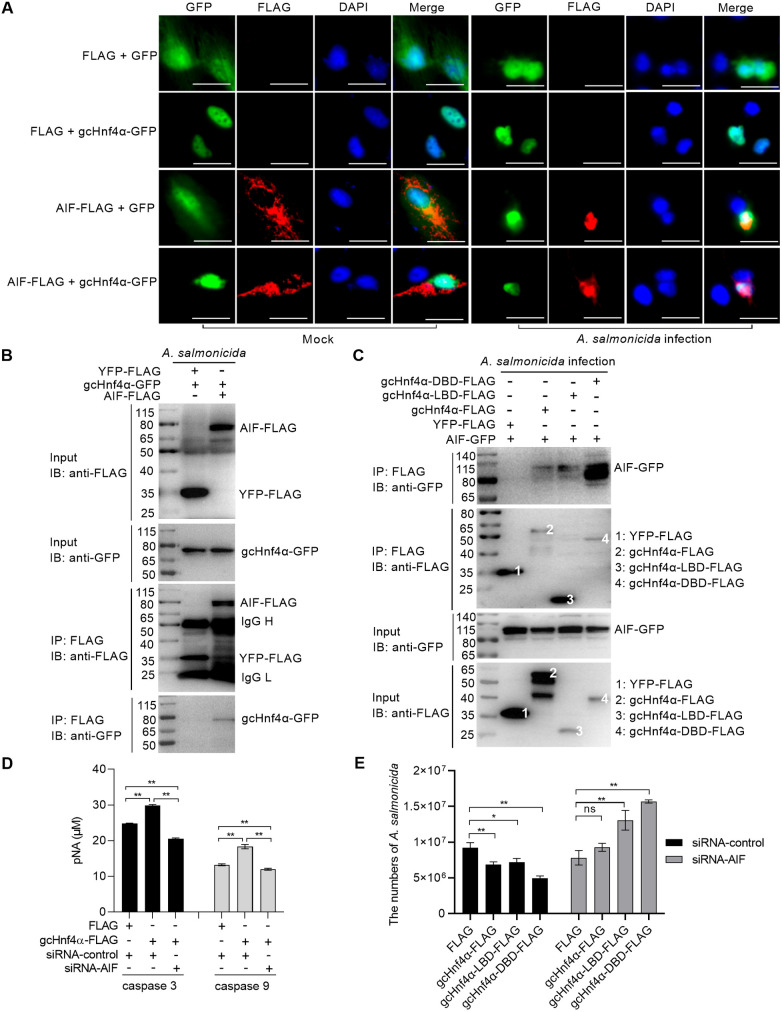
Grass carp Hnf4α depends on AIF to induce the activities of caspase 3 and caspase 9 for inhibiting pathogen proliferation. (A) Subcellular colocalization of gcHnf4α with AIF in *A. salmonicida*-infected CIK cells (MOI = 0.5, 6 hpi). Scale bar = 10 μM. (B-C) Co-immunoprecipitation confirms interaction between gcHnf4α (full-length/DBD) and AIF. (D-E) AIF knockdown reduces caspase 3/9 activity and enhances *A. salmonicida* proliferation in gcHnf4α-overexpressing cells. Data are mean ± SEM (n = 3). Statistical significance was determined by Student’s t-test (*p < 0.05, **p < 0.01; ns = not significant).

Previous research has established that AIF participates in both caspase-dependent and caspase-independent apoptotic pathways [37,38]. To determine whether AIF-mediated apoptosis is involved in the anti-pathogen function of gcHnf4α, we first investigated the interaction between gcHnf4α and AIF. In uninfected CIK cells, AIF was predominantly localized in the cytoplasm. However, upon *A. salmonicida* infection, AIF translocated to the nucleus. Significantly, during *A. salmonicida* infection, after AIF translocated to the nucleus, gcHnf4α co-localized with AIF in the nuclear compartment (Fig 5A). Co-immunoprecipitation assays confirmed a physical interaction between gcHnf4α and AIF (Fig 5B). Further analysis revealed that gcHnf4α interacted with AIF through both its LBD and DBD, with a more prominent interaction via the DBD (Fig 5C).

To explore the relationship between the caspase-dependent pathway and the AIF -mediated pathway in the gcHnf4α-induced activation of caspase 3 and caspase 9, we knocked down AIF expression and then measured the enzymatic activities of caspase 3 and caspase 9 in CIK cells transfected with gcHnf4α-FLAG or the FLAG control. Our published work validated that siRNA-AIF-3 efficiently knocks down AIF expression (27). Caspase activity assays revealed that AIF silencing significantly impaired gcHnf4α-induced activation of caspase 3 and caspase 9 during both *A. salmonicida* infection (Fig 5D) and GCRV infection (S4C Fig). Additionally, AIF knockdown abrogated gcHnf4α-mediated restriction of *A. salmonicida* proliferation and GCRV replication (Figs 5E, S4D).

Collectively, these findings suggest that gcHnf4α relies on AIF to activate caspase 3 and caspase 9, thereby inhibiting pathogen proliferation.

### gcHnf4α can bind to caspase 3 promoter HNF4A motif rather than AIF or caspase 9 promoter HNF4A motif

To characterize the sequence-specific DNA-binding activity of gcHNF4α at the promoters of apoptosis-related genes, we performed chromatin immunoprecipitation (ChIP) assays using FLAG-tagged gcHNF4α (gcHNF4α-FLAG) in CIK cells, both under basal conditions and following *A. salmonicida* infection. Bioinformatics analysis of 5’ upstream regulatory regions identified two putative HNF4A consensus motifs (VAAABBKVM; V = A/C/G, B = T/C/G, K = T/G, M = A/C) in the *aif* promoter (positions -716/-708 and -1679/-1671 relative to transcription start site), two in the *caspase 3* promoter (positions -1142/-1128 and -214/-201 relative to transcription start site), and one in the *caspase 9* promoter (-67/-53 relative to transcription start site) (S5 Fig).

ChIP RT-PCR analysis revealed significant enrichment of the caspase 3 promoter at HNF4α Site 2 (-214/-201 region) in gcHnf4α-FLAG immunoprecipitates compared to IgG controls, both under normal culture conditions and during bacterial challenge. In contrast, no specific binding was detected at either HNF4A motif in the *aif* promoter or the single motif in the *caspase 9* promoter (S6 Fig).

These findings demonstrate that gcHnf4α exhibits highly specific promoter occupancy at the *caspase 3* locus, selectively engaging the downstream HNF4A motif to facilitate direct transcriptional regulation.

### gcHnf4α induces early apoptosis in an AIF-, caspase 3- and caspase 9-dependent manner

**Fig 6:**
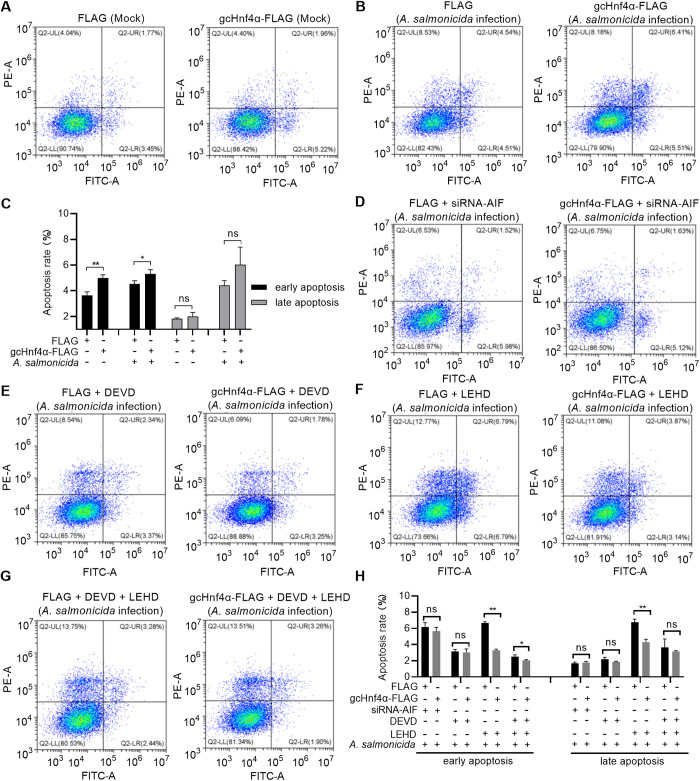
Grass carp Hnf4α induces early apoptosis dependent of AIF, caspase 3 and caspase 9. (A-C) gcHnf4α overexpression induces early apoptosis in CIK cells with/without *A. salmonicida* infection. CIK cells were transfected with gcHnf4α-FLAG or empty vector for 36 h, infected at MOI = 0.5, and harvested at 6 hpi for apoptosis assays using Annexin V-FITC/PI double staining. (D-H) Functional dependency of gcHnf4α-mediated apoptosis on AIF, caspase 3, and caspase 9. (D) AIF was knocked down using siRNA-AIF, (E and F) caspase 3/9 were inhibited with Z-DEVD-FMK (50 μM) or Z-LEHD-FMK (50 μM), and (G) combined inhibition was performed for 1 h prior to *A. salmonicida* infection (MOI = 0.5). Apoptosis was quantified at 6 hpi via flow cytometry, distinguishing early (Annexin V + /PI-) and late (Annexin V + /PI+) apoptotic cells. Data are mean ± SEM (n = 3). Statistical significance was determined by Student’s t-test (*p < 0.05, **p < 0.01; ns = not significant).

To investigate the pro-apoptotic function of gcHnf4α and its dependency on downstream effectors, we performed gain-of-function studies in CIK cells transfected with gcHnf4α-FLAG or empty vector, followed by mock infection or challenge with *A. salmonicida*. Flow cytometry using Annexin V-FITC/PI double staining revealed that gcHnf4α overexpression significantly increased early apoptotic cells in uninfected cells and in infected cells compared to controls, with no significant change in late apoptotic/necrotic cells (Fig 6A-6C).

To dissect the molecular mechanisms, we employed siRNA-mediated silencing of AIF and pharmacological inhibition of caspase 3 (Z-DEVD-FMK, 50 μM) and caspase 9 (Z-LEHD-FMK, 50 μM). AIF knockdown abrogated gcHnf4α-induced early apoptosis, reducing Annexin V+ cells to control levels (Fig 6D and 6H). Inhibition of caspase 3 with Z-DEVD-FMK completely blocked early apoptosis induced by gcHnf4α (Fig 6E and 6H), confirming its critical role in the execution phase. Notably in the case of caspase 9 activity inhibition, overexpression of gcHnf4α suppressed both early and late apoptosis (Fig 6F and 6H). During the co-treatment with the Z-DEVD-FMK and Z-LEHD-FMK, the overexpression of gcHnf4α slightly inhibited early apoptosis (Fig 6G and 6H).

Collectively, these findings demonstrate that gcHnf4α induces early apoptosis in a manner dependent on AIF, caspase 3, and caspase 9.

### Caspase 3 and caspase 9 are essential effectors of gcHnf4α-mediated host defense against bacterial, viral, and coinfection challenges

**Fig 7:**
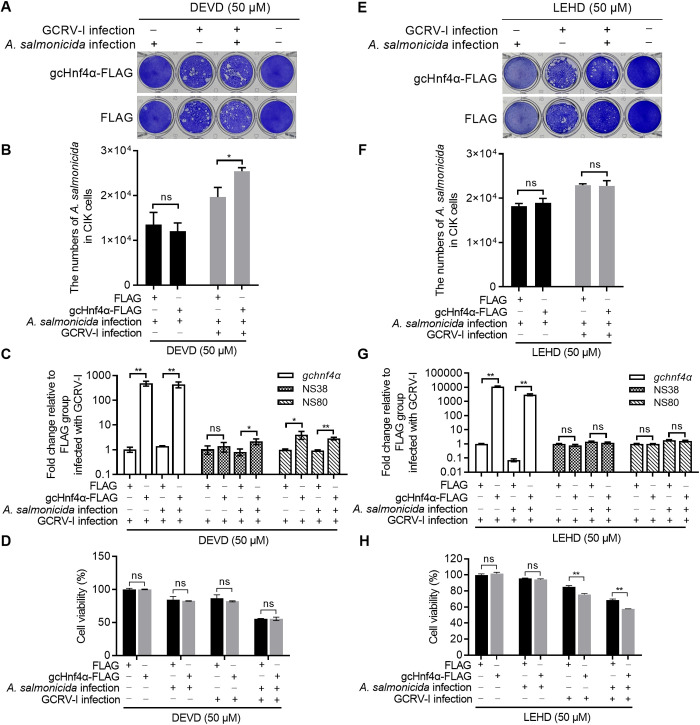
Caspase inhibition abolishes gcHnf4α-mediated protection *in vitro.* (A-D) The effect of caspase 3 inhibition on gcHnf4α-mediated cell survival (A), *A. salmonicida* proliferation (B), viral gene expression (C), and cell viability (D), with corresponding analyses for caspase 9 inhibition (E-H). CIK cells transfected with gcHnf4α-FLAG or empty vector were pre-treated with 50 μM Z-DEVD-FMK (caspase 3) or 50 μM Z-LEHD-FMK (caspase 9) for 1 h, then infected with *A. salmonicida* (MOI = 0.5), GCRV-I (MOI = 0.5), or coinfected. These cells were harvested at 24 hpi for crystal violet staining (A, E), plate colony-counting for bacterial proliferation (B, F), qRT-PCR for viral gene expression (C, G), and CCK-8 assay for cell viability (D, H). Data are mean ± SEM (n = 3). Statistical significance was determined by Student’s t-test (*p < 0.05, **p < 0.01; ns = not significant).

To determine the requirement for caspase 3 and caspase 9 in gcHnf4α-dependent protective immunity, we performed loss-of-function studies using pharmacological inhibitors in *in vitro* CIK cell models and *in vivo* zebrafish larvae challenged with *A. salmonicida*, GCRV-I (*in vitro* CIK cell models) or GCRV-II (*in vivo* zebrafish model), or their coinfection. Pharmacological inhibition of caspase 3 or caspase 9 abrogated the protective effects of gcHnf4α. Compared with CIK cells transfected with FLAG, overexpression of gcHnf4α in the case of inhibition of caspase 3 activity by Z-DEVD-FMK failed to inhibit or even promote the proliferation of *A. salmonicida* during *A. salmonicida* infection and *A. salmonicida* associated GCRV-I coinfection (Fig 7A and 7B). The expression of NS38 and NS80 of GCRV-I during GCRV-I infection and *A. salmonicida* associated GCRV-I coinfection remained unchanged or was increased by overexpression of gcHnf4α in the case of inhibition of caspase 3 activity by Z-DEVD-FMK when compared with CIK cells transfected with FLAG (Fig 7A and 7C). Importantly, overexpression of gcHnf4α in the case of inhibition of caspase 3 activity by Z-DEVD-FMK had no obvious effect on cell survival compared with the control group transfected with FLAG during *A. salmonicida* infection, GCRV-I infection, and *A. salmonicida* associated GCRV-I coinfection (Fig 7D). Compared with CIK cells transfected with FLAG, overexpression of gcHnf4α in the case of inhibition of caspase-9 activity by Z-LEHD-FMK inhibitor had no obvious effects on the proliferation of *A. salmonicida* during *A. salmonicida* infection and *A. salmonicida* associated GCRV-I coinfection (Fig 7E and 7F), the expression of viral genes during GCRV-I infection and *A. salmonicida* associated GCRV-I coinfection (Fig 7E and 7G), and cell survival during *A. salmonicida* infection (Fig 7H). The cell survival during GCRV-I infection and *A. salmonicida* associated GCRV-I coinfection was even impaired by overexpression of gcHnf4α in the case of inhibition of caspase-9 activity (Fig 7H). These data suggest that the inhibition of caspase 3 or caspase 9 abolished the *in vitro* protective function of gcHnf4α during *A. salmonicida* infection, GCRV-I infection, and *A. salmonicida* associated GCRV-I coinfection.

**Fig 8:**
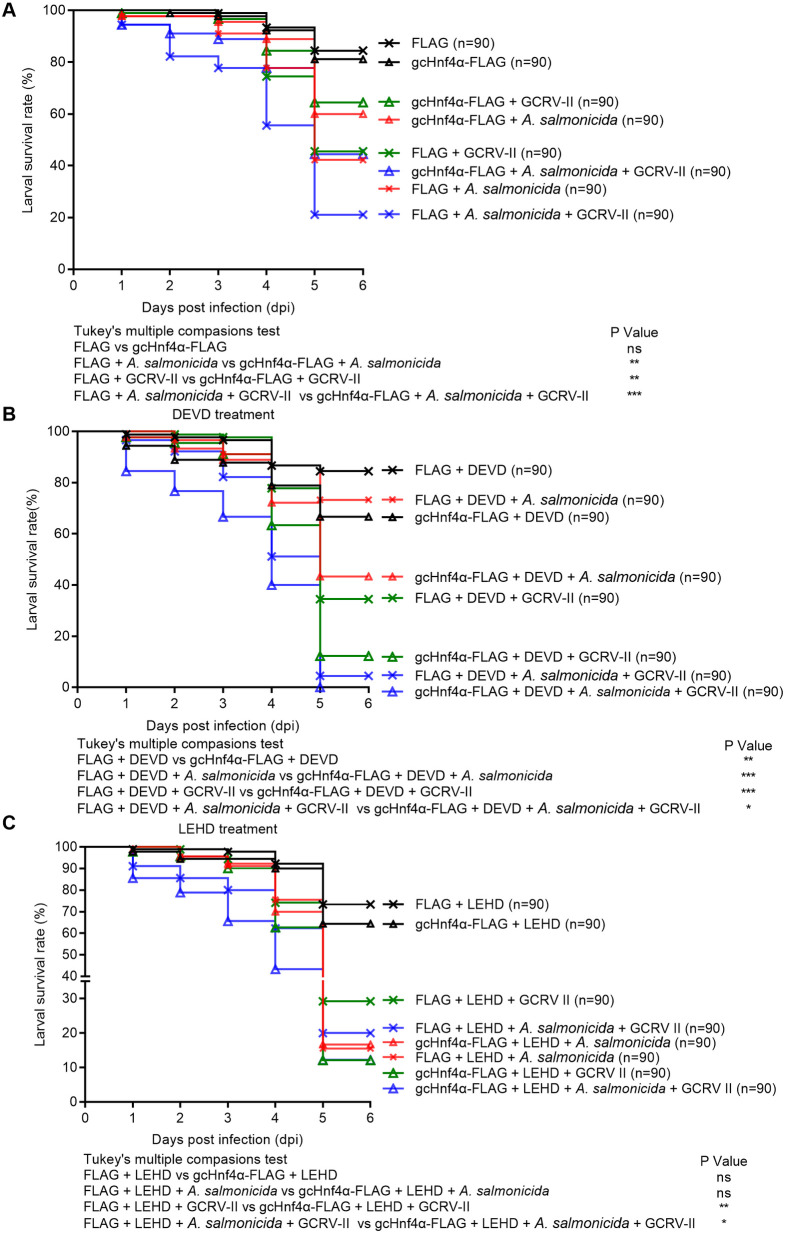
Caspase inhibition abolishes gcHnf4α-mediated protection *in vivo.* (A) gcHnf4α overexpression enhances survival of zebrafish larvae infected with *A. salmonicida*, GCRV-II, or coinfected with both pathogens. Larvae microinjected at 4 dpf with FLAG or gcHnf4α-FLAG (n = 90/group) were infected with 1 × 10⁷ CFU/mL *A. salmonicida*, 2.76 × 10¹⁰ copies/mL GCRV-II, or a mixture of both pathogens. Mortality was recorded daily from 1–6 dpi. (B-C) Caspase 3 (B) or caspase 9 (C) inhibition attenuates gcHnf4α-mediated protection against infection. Larvae injected with FLAG or gcHnf4α-FLAG were pre-treated with 75 μM Z-DEVD-FMK (caspase 3 inhibitor) or Z-LEHD-FMK (caspase 9 inhibitor) for 12 h prior to infection (n = 90/group). Survival was monitored daily from 1–6 dpi. Significance in survival curves (A-C) was determined by Log-Rank (Mantel-Cox) test.

GCRV-I is associated with a long latent period and low mortality, and differs from GCRV-II, which is the current popular and fatal strain in aquaculture. Here, we used GCRV-II rather GCRV-I to infect zebrafish. To evaluate the *in vivo* function of gcHnf4α, zebrafish larvae microinjected with FLAG or gcHnf4α-FLAG were infected with *A. salmonicida* or GCRV-II, or coinfected with *A. salmonicida* and GCRV-II. Overexpression of gcHnf4α had no obvious effect on larvae survival in the case of uninfected condition. For zebrafish larvae microinjected with FLAG, *A. salmonicida*, GCRV-II or *A. salmonicida* associated GCRV-II coinfection impaired larvae survival, with the decreased survival rate by 42.22%, 38.88%, 63.33% respectively. gcHNF4α overexpression increased survival by 17.88% (*A. salmonicida*), 18.88% (GCRV-II), and 23.33% (coinfection) compared to FLAG controls (Fig 8A). Z-DEVD-FMK treatment completely abrogated this protection mediated by gcHnf4α, with the decreased survival rates by 30% (*A. salmonicida*), 22.22% (GCRV-II), and 7.78% (coinfection) compared with the inhibitor-treated FLAG controls (Fig 8B). Overexpression of gcHnf4α in the case of inhibition of caspase 9 had context-specific effects: no impact during *A. salmonicida* mono-infection, but a significant 15%/6% decrease in survival during GCRV-II/coinfection (Fig 8C), highlighting its specialized role in antiviral immunity. These data suggest that the inhibition of caspase 3 or caspase 9 abolished the *in vivo* protective function of gcHnf4α during *A. salmonicida* infection, GCRV-II infection, and *A. salmonicida* associated GCRV-II coinfection.

### Pathogen-pathogen synergism proceeds independently of Hnf4α, while zfHnf4α recapitulates gcHnf4α’s dual functions as a host-defense hub

To establish the genetic dependency of piscine *hnf4α*-mediated immunity on caspases, we generated *hnf4α*^−/−^ zebrafish using a CRISPR/Cas9 system targeting Exon 2 of the *hnf4α* locus. A 22-bp gRNA was designed to induce frameshift mutations. Sanger sequencing revealed a representative 2-bp insertion mutation (*zfhnf4α*^−/−^-2IS) leading to premature translation termination (S7A and S7B Fig). Western blot analysis using the above-mentioned anti-Hnf4α polyclonal antibody confirmed ablation of WT Hnf4α protein slightly larger than 50 kDa (matching the size of zfHnf4α-FLAG) in *zfhnf4α*^−/−^-2IS homozygous larvae (S2C and S7C Figs).

**Fig 9:**
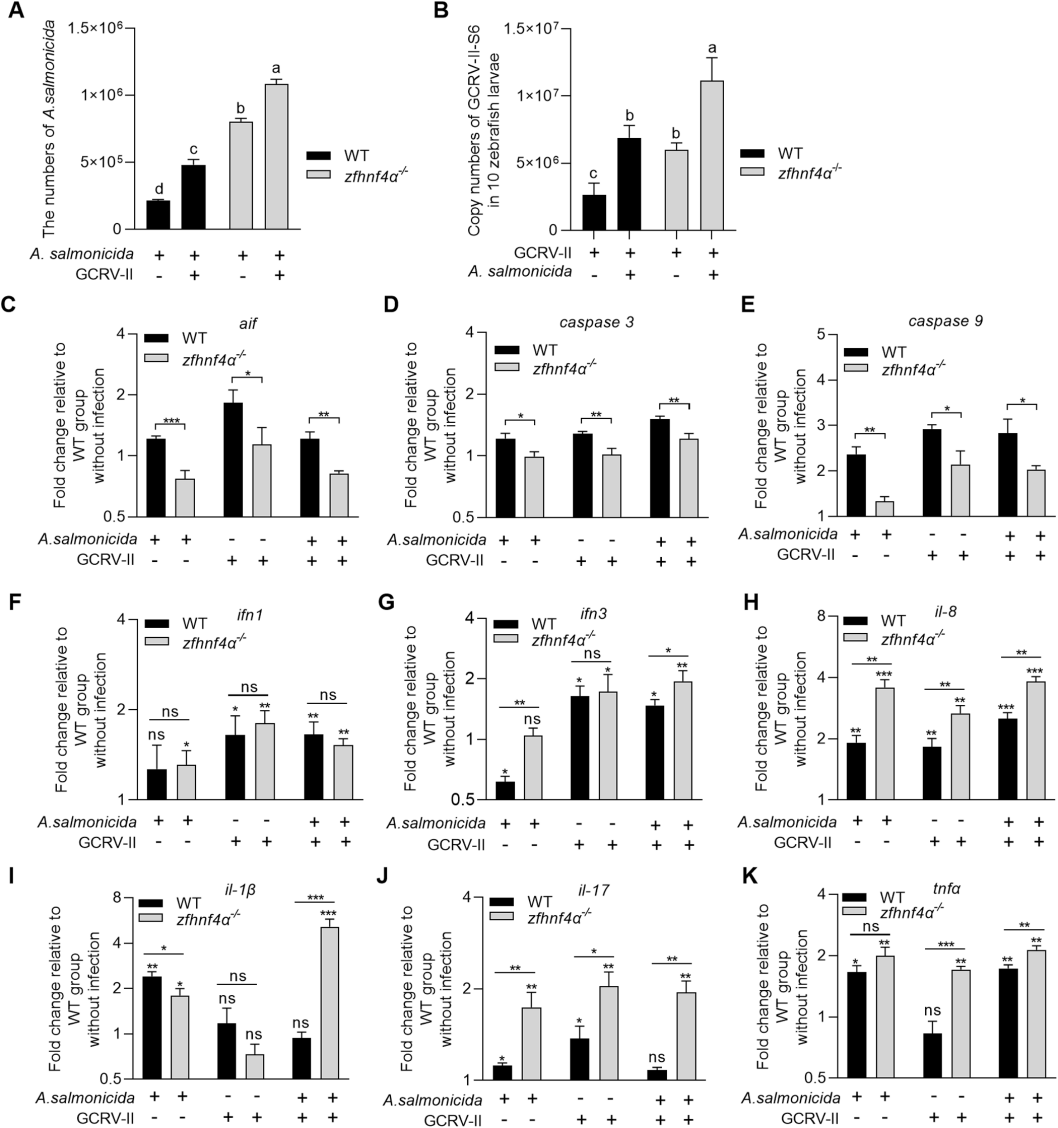
zfHnf4α restrains pathogen replication and modulates apoptotic and inflammatory gene expression during single and co-infections. (A) Impact of *zfhnf4α* knockout on *A. salmonicida* proliferation in single and GCRV-II co-infections. (B) Effect of *zfhnf4α* knockout on GCRV-II replication in single and *A. salmonicida* co-infections. (C-E) *zfhnf4α* knockout-mediated changes in *aif*, *caspase 3*, and *caspase 9* expression during single (*A. salmonicida* or GCRV-II) and co-infections. (F-K) *zfhnf4α*-mediated regulation of interferons (*ifn1* and *ifn3*) and pro-inflammatory cytokines (*il-8*, *il-1β*, *il-17*, **tnf*α*) expression across infection contexts. WT or *zfhnf4α*^⁻/⁻^ larvae were infected with *A. salmonicida* (1 × 10⁷ CFU/mL), GCRV-II (2.76 × 10¹⁰ copies/mL), or their mixture. At 48 hpi, larvae were analyzed by colony counting for bacterial proliferation or qRT-PCR for expression levels including viral gene, interferons and pro-inflammatory cytokines. Data are mean ± SEM (n = 3). Statistical significance was determined by Student’s t-test (*p < 0.05, **p < 0.01; ***p < 0.001, ns = not significant).

To dissect whether pathogen-pathogen interactions (e.g., GCRV-induced modulation of *A. salmonicida* proliferation) depend on *zfhnf4α* and whether *zfhnf4α* has a function similar to that of *gchnf4α*, we leveraged *zfhnf4α* knockout larvae to compare pathogen dynamics across single and co-infection scenarios, while paralleling analyses of transcriptional regulation of *aif*, *caspase 3* or *caspase 9*. In pathogen interaction assays, *zfhnf4α* deficiency significantly enhanced *A. salmonicida* proliferation in both single infection and *A. salmonicida*-GCRV-II co-infection; critically, bacterial loads remained significantly higher in co-infection versus matched single-infection groups regardless of genotype (wild-type vs. *zfhnf4α* ⁻ /⁻) (Fig 9A). Similarly, *zfhnf4α* knockout augmented GCRV-II replication in single and co-infections, with viral copy numbers consistently elevated in co-infection relative to single-infection conditions across both genetic backgrounds (Fig 9B). These findings indicate that the synergistic enhancement of pathogen replication during co-infection proceeds independently of *zfhnf4α*.

Concurrent analyses of apoptotic gene regulation revealed that *zfhnf4α*, like its grass carp ortholog, transcriptionally controls core apoptotic effectors: qRT-PCR showed *zfhnf4α* deficiency significantly reduced infection-induced mRNA levels of *aif*, *caspase 3*, and *caspase 9* in both single (*A. salmonicida* or GCRV-II) and co-infection models (Fig 9C-9E). This mirrors *gchnf4α*’s role in regulating *aif*, *caspase 3*, and *caspase 9*, confirming evolutionary conservation of *hnf4α*-mediated transcriptional control over apoptotic machinery in teleosts.

Given the functional conservation of *hnf4α* between zebrafish and grass carp in *A. salmonicida*/GCRV-II single and co-infections, we used *zfhnf4α* knockout larvae to explore whether *zfhnf4α* modulates antimicrobial responses via interferons or inflammatory cytokines. qRT-PCR analyses revealed distinct regulatory patterns (Fig 9F-9K). In wild-type zebrafish, *A. salmonicida* infection repressed *ifn3* but upregulated pro-inflammatory cytokines (*il-8*, *il-1β*, *il-17*, **tnf*α*); GCRV-II infection induced *ifn1*, *ifn3*, *il-8*, and *il-17*; and co-infection upregulated *ifn1*, *ifn3*, *il-8*, and **tnf*α*. Notably, *zfhnf4α* knockout exerted context-dependent effects: in *A. salmonicida* single infection, it significantly upregulated *ifn3*, *il-8*, and *il-17* but downregulated *il-1β* relative to wild-type; in GCRV-II single infection, knockout did not affect *ifn1*, *ifn3*, or *il-1β* but enhanced *il-8*, *il-17*, and **tnf*α* expression; and in co-infection, knockout upregulated *ifn3* and all tested pro-inflammatory cytokines (*il-8*, *il-1β*, *il-17*, **tnf*α*). These findings indicate that interferon regulation during GCRV-II infection is primarily pathogen-driven with minimal *zfhnf4α* involvement, *zfhnf4α* selectively suppresses *A. salmonicida*-induced il-1β, and *zfhnf4α* deficiency amplifies pro-inflammatory cytokine induction across all infection contexts.

### Caspase 3 and caspase 9 rescue Hnf4α-deficient survival deficits during bacterial, viral, and coinfection challenges

**Fig 10:**
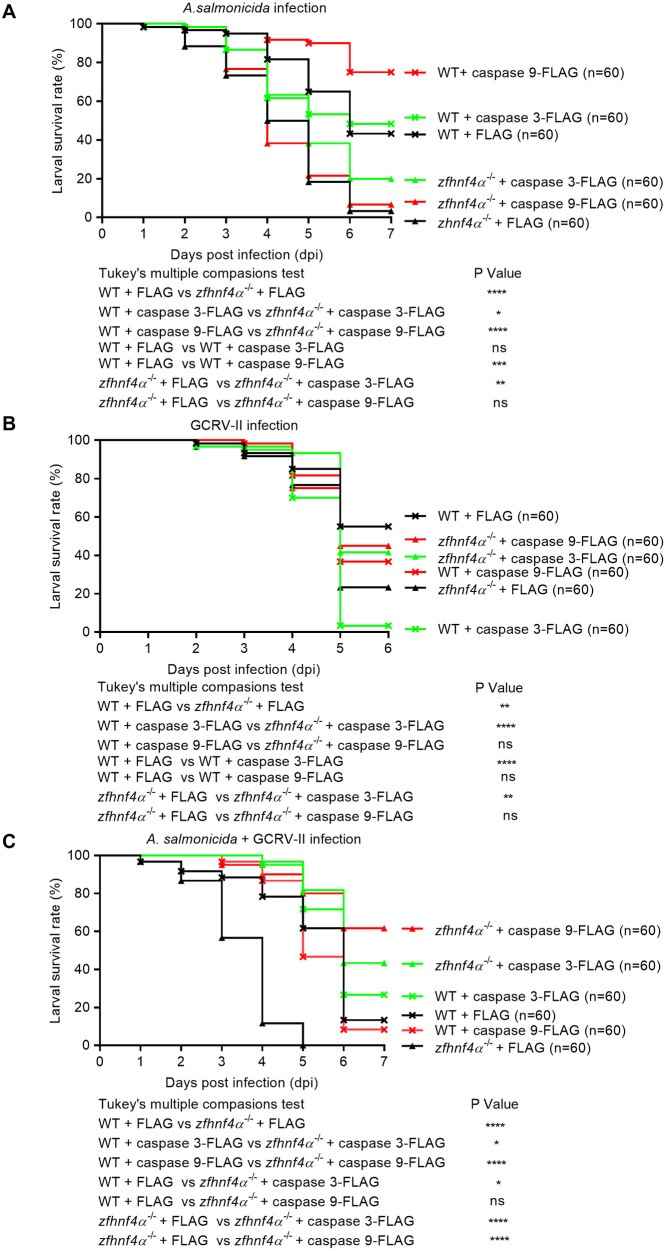
Overexpression of caspase 3 or caspase 9 rescues impaired survival of *zfhnf4α*^−^^/^^−^ larvae infected with *A. salmonicida* (A), GCRV-II (B), or coinfected with both pathogens (C). Wild-type (WT) or *zfhnf4α*^−/−^ larvae were microinjected at 4 dpf with FLAG, caspase 3-FLAG, or caspase 9-FLAG, then infected with 1 × 10⁷ CFU/mL *A. salmonicida*, 2.76 × 10¹⁰ copies/mL GCRV-II, or a mixture of both (n = 60/group). Survival curves were analyzed for significance using the Log-Rank test.

During *A. salmonicida* challenge, *zfhnf4α*^−/−^-2IS zebrafish exhibited severe susceptibility, with only 3.33% survival by 7 dpi—markedly lower than 43.33% survival in WT controls transfected with empty vector (Fig 10A). Overexpression of caspase 3 in *zfhnf4α*^−/−^-2IS larvae restored survival to 20.0% (16.67% increase), whereas caspase 9 overexpression had no rescue effect despite enhancing WT survival by 31.67%. These results indicate caspase 3 is a downstream effector of Hnf4α in antibacterial immunity, while caspase 9’s protective function requires intact Hnf4α signaling.

Against GCRV-II infection, *zfhnf4α*^−/−^-2IS showed a 31.67% survival deficit compared to WT (23.33% vs. 55.0% at 6 dpi). Strikingly, caspase 3/9 overexpression had opposing effects in WT vs. knockout zebrafish. In WT zebrafish larvae, caspase 3/ caspase 9 overexpression reduced survival by 51.67%/18.33%, indicating excess caspase activity is detrimental to host. In *zfhnf4α*^−/−^-2IS larvae, caspase 3/ caspase 9 overexpression rescued survival to 41.67%/45.0%, respectively (18.34%/21.67% increases, Fig 10B). These data demonstrate caspase 3/9 act as essential mediators of Hnf4α-dependent antiviral immunity, likely by balancing apoptotic signaling to restrict viral replication.

During *A. salmonicida*-GCRV-II coinfection, *zfhnf4α*^−/−^-2IS larvae showed 100% mortality by 5 dpi, whereas WT controls transfected with empty vector had 13.33% survival (Fig 10C). Caspase overexpression revealed distinct rescue patterns: caspase 3 enhanced WT survival by 13.34% and *zfhnf4α*^−/−^-2IS survival by 43.33%, highlighting its critical role in dual-pathogen defense; caspase 9 improved *zfhnf4α*^−/−^-2IS survival by 61.67% (from 0% to 61.67%) but had no effect in WT, suggesting a specialized role in compensating for Hnf4α loss during complex infections.

## Discussion

HNF4α, a nuclear transcription factor, is essential for regulating liver bile acid signaling and chronic liver disease progression [7,39]. While mammalian HNF4α is well-documented to bind overlapping sequences in the HBV enhancer II to promote viral core promoter activity and HBV replication [40,41], its role in bacterial infection and bacterial-viral coinfection has remained unclear. This study demonstrates that piscine HNF4α contributes to host antibacterial and antiviral defense by inducing the expression and activities of caspase 3 and caspase 9, positioning it as a potential therapeutic target for bacterial, viral, and coinfection scenarios.

The dynamic expression of gcHnf4α—upregulated at 6 and 24 hpi, yet downregulated at 48 hpi—reflects a spatiotemporally tuned host response. The dynamic, counterbalanced expression of *gchnf4α* and *gchnf4β*—characterized by *gchnf4α* peaking at 6 hpi then declining by 48 hpi, while *gchnf4β* exhibits inverse kinetics [27]—represents a sophisticated, phase-specific immune adaptation in teleosts. This temporal partitioning likely reflects an evolutionary trade-off: the early upregulation of *gchnf4α* may prioritize rapid antibacterial and antiviral defenses (e.g., caspase activation, viral inclusion body disruption), while its late downregulation prevents excessive apoptosis and metabolic collapse. Concurrently, *gchnf4β*’s delayed induction at 48 hpi could compensate for *gchnf4α*’s decline, maintaining immune surveillance via AIF-dependent pathways [27]. This functional handoff is further supported by genetic epistasis in zebrafish: while *hnf4β* ⁻ ^/^ ⁻ mutants develop normally, combined *hnf4α* ⁻ */* ⁻ ;*hnf4β ⁻ *^*/*^⁻ or *hnf4α* ⁻ / ⁻ ;*hnf4β* ⁻ / ⁻ ;*hnf4γ* ⁻ / ⁻ knockouts exhibit catastrophic embryonic defects [28]. These data underscore critical, cooperative roles for Hnf4α and Hnf4β in early development and pathogen infection, likely through redundant regulation of metabolic and immune programs [21,28]. This dynamic interplay highlights a teleost-specific strategy to balance pathogen control with metabolic homeostasis. Future studies should dissect the precise molecular switches governing this transition and explore whether pharmacological modulation of Hnf4α/β dynamics could enhance disease resistance in aquaculture.

Previous studies have shown that *Caenorhabditis elegans* NHR-86, a mammalian HNF4 homolog, is not required for basal resistance to *Pseudomonas aeruginosa* but can be activated by immunostimulatory small molecules to drive a protective transcriptional program against bacterial infection [42]. Sepsis induces progressive hepatic loss of HNF4α function, and the HNF4α agonist NCT protects against sepsis independently of bacterial load, highlighting HNF4α’s critical role in sepsis tolerance [43]. Additionally, long-term oral administration of the HNF4α agonist N-trans-caffeoyltyramine (NCT) prevents hepatic steatosis [44]. *A. salmonicida*, a primary fish pathogen causing bacterial septicemia, and GCRV, which induces severe hemorrhagic disease in grass carp, pose significant challenges to aquaculture [45,46]. Our study reveals that piscine Hnf4α regulates cell survival, viral gene expression, and bacterial proliferation during *A. salmonicida* infection, GCRV-I infection, and coinfection. *In vivo* assays showed that gcHnf4α overexpression significantly improved larval survival during *A. salmonicida*, GCRV-II, or coinfection, while *hnf4α* knockout led to drastically reduced survival, confirming Hnf4α’s critical protective role across infection types. These findings highlight Hnf4α as a promising therapeutic target for aquacultural diseases such as bacterial septicemia, grass carp hemorrhagic disease, and their coinfections, while revealing a key adaptive specialization: unlike mammalian Hnf4α, which promotes viral replication [47–49], teleost Hnf4α acts as a pathogen restrictor. This functional shift stems from structural divergence—teleost-specific substitutions in LBD residues reconfigure protein interaction interfaces, enabling novel antiviral functions. This specialization likely evolved in response to the unique selective pressures of aquatic environments, where teleosts face persistent co-infections by bacteria and viruses. Unlike mammals, which rely on adaptive immunity to combat acute infections, teleosts depend heavily on innate responses. gcHnf4α’s dual role in activating apoptosis (via caspase 3/9) and restricting pathogen replication (via VIBs inhibition) represents a streamlined, cost-effective strategy to counteract polymicrobial threats. The conservation of this mechanism in zebrafish further supports its evolutionary significance as a teleost-specific adaptation.

Apoptotic pathways, categorized as intrinsic and extrinsic, play pivotal roles in disease pathogenesis. The extrinsic pathway activates caspase 8, whereas the intrinsic (mitochondrial) pathway involves Bax/Bcl-2-mediated release of cytochrome c and AIF from mitochondria, activating caspase 9 or inducing caspase-independent nuclear apoptosis via AIF translocation [50–52]. Caspase 3, a key apoptosis executor, is activated by both caspase 8 and 9. Mammalian HNF4 comprises HNF4α (NR2A1) and HNF4γ (NR2A2) subtypes encoded by HNF4α and HNF4γ [53]. In colorectal cancer cells, HNF4γ knockdown promotes caspase-dependent apoptosis via the intrinsic pathway, activating caspase 9 followed by caspase 3 [54]. Here, gcHnf4α overexpression enhanced caspase 3/9 expression and activity during infections, inducing early apoptosis dependent on AIF, caspase 3, and caspase 9. Notably, piscine Hnf4α induces apoptosis via the intrinsic pathway, contrasting with mammalian HNF4γ’s role in inhibiting intrinsic pathway-mediated apoptosis. Interesting, the concurrent upregulation of pro- and anti-apoptotic genes by gcHnf4α overexpression likely reflects a nuanced regulatory strategy to fine-tune apoptotic responses, avoiding unchecked cell death (which would compromise tissue integrity) while retaining pathogen-clearing capacity. This balance is biologically critical: pro-apoptotic genes drive elimination of infected cells, while anti-apoptotic genes prevent excessive hepatocyte loss (vital for liver function in grass carp). Similar dual regulation is observed in mammalian p53 or NF-κB signaling, where concurrent activation of opposing effectors enables context-dependent cellular outcomes [55–58]. In pathogen infection, this dynamic may ensure apoptosis is constrained to pathogen-containing cells, sparing healthy tissue—aligning with gcHnf4α’s role as a metabolic-immune hub balancing defense and homeostasis.

Apoptogenic factors like cytochrome c, procaspases 2/3/9, and AIF are mitochondrial intermembrane proteins. AIF, upon death signaling, translocates to the nucleus to induce chromatin condensation and fragmentation in a caspase-independent manner [59]. In normal CIK cells, AIF localized primarily to the cytoplasm, whereas infection triggered nuclear translocation. Caspase 3 and 9 exhibited both cytoplasmic and nuclear distribution in infected cells. Knockdown of AIF or inhibition of caspase 3/9 completely blocked gcHnf4α-induced apoptosis. AIF knockdown additionally abolished gcHnf4α-induced caspase 3/9 activity, even reducing basal activity below control levels. These results indicate that gcHnf4α-mediated apoptosis occurs via both caspase- and AIF-dependent mechanisms.

**Fig 11:**
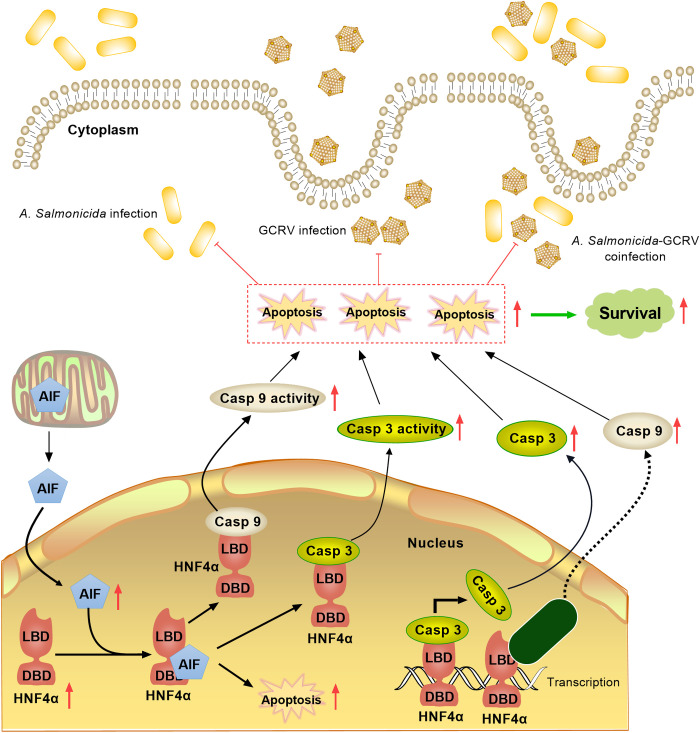
A schematic model of the role of piscine Hnf4α targeting AIF and caspase 3/9 signaling in inhibiting the infection of *A. salmonicida* and GCRV as well as their co-infection. Teleost Hnf4α acts as a dual-action immune regulator, activating a conserved AIF-caspase 3/9 apoptotic pathway to restrict pathogens in single and co-infections: its DBD interacts with AIF to induce caspase-independent nuclear apoptosis, while its LBD engages caspases 3/9 for canonical apoptotic signaling. Furthermore, gcHnf4α directly transactivates caspase 3 through promoter binding and indirectly upregulates caspase 9 via transcriptional crosstalk. This mechanism underscores piscine Hnf4α’s role as a signaling hub, integrating nuclear receptor activity with apoptotic machinery to counteract polymicrobial infection.

Mechanistically, piscine Hnf4α employs a bifunctional molecular scaffold to coordinate anti-pathogen immunity, as revealed by co-immunoprecipitation assays demonstrating direct physical association between gcHnf4α and the mitochondrial apoptotic trio (AIF, caspase 3, caspase 9). In *C. elegans*, the LBD of NHR-86/HNF4 senses pathogen-derived metabolites to activate protective transcription [60]. Mammalian HNF4’s LBD is critical for dimerization and coregulator interaction, while its DBD undergoes post-translational modifications (e.g., PRMT1-mediated methylation) to modulate DNA binding [3,61–64]. Notably, AIF interacted strongly with gcHnf4α’s DBD and weakly with its LBD. AIF knockdown abrogated gcHnf4α (full-length, DBD, and LBD)-mediated pathogen growth inhibition. Caspase 3/9 inhibition selectively impaired gcHnf4α full-length and LBD (but not DBD) function. Furthermore, gcHnf4α also directly transactivates caspase 3 via promoter binding, highlighting the conserved function of Hnf4α in transcriptional regulation. Although gcHnf4α does not bind directly to the caspase 9 promoter, its overexpression elevates caspase 9 expression. The direct binding of gcHnf4α to the caspase 3 promoter implies a similar regulatory logic may apply to upstream transcription factors of caspase 9, forming a transcriptional hierarchy. Thus, the indirect upregulation of caspase 9 by gcHnf4α likely arises from a combinatorial network of transcriptional crosstalk rather than direct promoter engagement. Notably, the functional dependency on AIF and caspase 3/9 supports a “transcription-post-translational synergy mechanism”: gcHnf4α transcriptionally upregulates caspase 3 and recruits AIF/caspase 9/caspase 3 via protein interactions to form signaling complexes that drive apoptotic execution (Fig 11). This mechanism underscores gcHnf4α’s role as a signaling hub, integrating nuclear receptor activity with apoptotic machinery. Elucidating these mechanisms would deepen our understanding of how nuclear receptors coordinate complex immune responses of relevance to aquatic pathogen control.

In conclusion, this study uncovers a missing link in teleost immune evolution: unlike mammalian HNF4, which primarily regulates metabolism and oncogenesis, piscine Hnf4α has evolved to integrate apoptosis and transcription into a unified anti-pathogen program. Given its protective role across bacterial, viral, and coinfection models, Hnf4α represents a promising therapeutic target for infectious disease management in aquaculture. However, this study is not without limitations. This study reliance on CIK cell lines and zebrafish models, while informative, may not fully recapitulate the physiological complexity of grass carp *in vivo*; thus, validation in primary grass carp hepatocytes and grass fish *in vivo* remains critical. Additionally, the role of Hnf4α ligands (e.g., bile acids) in modulating its antiviral and antibacterial functions remains unaddressed, leaving gaps in understanding contextual immune tuning. Future work will focus on: (i) *in vivo* grass carp infection models to dissect gcHnf4α’s tissue-specific roles; (ii) screening for Hnf4α agonists (e.g., natural bile acid derivatives) to enhance anti-pathogen-directed immune responses; and (iii) exploring cross-talk between gcHnf4α and other immune hubs in polymicrobial infections. These works will advance gcHnf4α as a target for aquacultural disease control.

## Materials and methods

### Ethics statement

All animal experiments were conducted in accordance with the Guide for the Care and Use of Laboratory Animals and approved by the Institute of Hydrobiology, Chinese Academy of Sciences (IHB/LL/2023056).

### Cells, bacteria and viruses

*Ctenopharyngodon* *idellus* kidney (CIK) cells were cultured in MEM (Gibco) culture medium with 10% FBS. Bacterial strain used in this study was wild-type *Aeromonas salmonicida* (GenBank: OR214944.1), which was isolated and preserved by our laboratory. *A. salmonicida* were cultured in Brain-Heart Infusion Broth (BHI, BD Biosciences) with ampicillin. Grass carp reovirus genotype I (GCRV-873, GCRV-I) was propagated in CIK cells using MEM supplemented with 2% FBS. GCRV-II was obtained from Prof. Yaping Wang (Institute of Hydrobiology, Chinese Academy of Sciences).

### Abs and reagents

The polyclonal rabbit antibody against GCRV-873 strain NS38 was previously generated and characterized [65]. The zebrafish or grass carp Hnf4α polyclonal antibody was custom-produced by Dia-An Biotech (Wuhan, China) using a solid-phase peptide synthesis approach. The synthetic peptide corresponded to amino acids 434–454 of zfHnf4α and gcHnf4α (S1 Appendix), validated by HPLC-MS with >90% purity and sufficient yield for immunization. The peptide was conjugated to keyhole limpet hemocyanin (KLH) via MBS (m-maleimidobenzoyl-N-hydroxysuccinimide ester)-mediated cross-linking at neutral pH, forming a peptide-KLH covalent complex as the complete antigen. New Zealand white rabbits were immunized with this complex, and the resulting antiserum was affinity-purified using antigen-coupled CNBr-activated agarose (GE Healthcare).

Commercially available reagents included: caspase 9 polyclonal antibody (Proteintech, Cat No.10380-1-AP), anti-FLAG mouse monoclonal antibody (Sigma-Aldrich, #F3165), anti-pTurbo-GFP rabbit polyclonal antibody (Everogen, #AB513), anti-HA rabbit monoclonal antibody (Proteintech, #51064–2-AP), goat anti-mouse/rabbit IgG-HRP conjugates (Thermo Fisher Scientific), Alexa Fluor 594/488-conjugated secondary antibodies (Thermo Fisher Scientific), DAPI (Thermo Fisher Scientific), RNase-free DNase I (Thermo Fisher Scientific), RevertAid First Strand cDNA Synthesis Kit (Thermo Fisher Scientific), protease inhibitor cocktail (Thermo Fisher Scientific), FLAG immunoprecipitation kit (Sigma-Aldrich), TRIzol reagent (Invitrogen, #15596026), Annexin V-FITC/PI Apoptosis Detection Kit (Vazyme, #A221-02), CytoTox 96 LDH Cytotoxicity Assay (Promega, #G1780), Cell Counting Kit-8 (Beyotime, #C0038), caspase 3/8/9 activity assays (Beyotime, #C1116, #C1152, #C1158), and caspase inhibitors Z-DEVD-FMK (Selleck, #S7313) and Z-LEHD-FMK TFA (Selleck, #S7312).

### Plasmid construction and sequence analysis

Plasmids including pTurbo-GFP (Evrogen), pcDNA3.1-FLAG-HA (Invitrogen), and p3 × FLAG-CMV-14 (Sigma-Aldrich) were maintained in our laboratory. gcHnf4α-FLAG, gcHnf4α-LBD-FLAG, gcHnf4α-DBD-FLAG, caspase 3-FLAG, and caspase 9-FLAG constructs were generated by PCR amplification using primer pairs gcHnf4α-F/ gcHnf4α-R, gcHnf4α-LBD-F/ gcHnf4α-LBD-R, gcHnf4α-DBD-F/ gcHnf4α-DBD-R, caspase 3-F/ caspase 3-R, and caspase 9-F/ caspase 9-R, respectively, and cloned into the p3 × FLAG-CMV-14 vector. gcHnf4α-GFP and caspase 3-GFP were constructed using primer pairs gcHnf4α-F1/ gcHnf4α-R1 and caspase 3-F1/ caspase 3-R1, respectively, and cloned into the pTurbo-GFP-N vector. Caspase 9-HA was generated using primer pair caspase 9-F2/ caspase 9-R2 and cloned into the pcDNA3.1-FLAG-HA vector. Primer sequences are provided in S2 Table. Transfections were performed in CIK cells using Neofect DNA transfection reagent.

Multiple sequence alignment of Hnf4α proteins was performed using Clustal Omega (https://www.ebi.ac.uk/jdispatcher/msa/clustalo). Phylogenetic analysis was conducted in MEGA 11, with a bootstrap value of 1000 to assess branch reliability. The Sequence Similarity Analysis was performed online (https://www.vectorbuilder.cn/tool/sequence-alignment.html).

### Constitutive and inducible expression of *gchnf4α* in wild-type grass carp

Healthy grass carp (mean weight 10 ± 1 g) were obtained from our laboratory and acclimatized in aerated freshwater at 25 ± 2°C for 1 week, fed a commercial pelleted diet at 3% body weight. For tissue distribution analysis, liver, heart, intestine, spleen, brain, gill, and kidney were collected from three untreated fish and stored at -80°C for qRT-PCR.

For inducible expression analysis, fish were randomized into four groups: a control group injected intraperitoneally with PBS (15 μL/g) and three experimental groups injected with *A. salmonicida* (1 × 10⁵ CFU/mL, 15 μL/g), GCRV-II (1.38 × 10⁹ copies/μL, 15 μL/g), or a mixture of *A. salmonicida* (1 × 10⁵ CFU/mL) and GCRV-II (1.38 × 10⁹ copies/μL) at 15 μL/g. Livers were collected from three fish per group at 6, 24, and 48 hpi.

### Immunofluorescence assays

To determine the effect of bacterial or viral infection on gcHnf4α subcellular localization, CIK cells seeded on coverslips in 24-well plates were transfected with gcHnf4α-FLAG or FLAG empty plasmid. At 24 h post-transfection, cells were infected with *A. salmonicida* or GCRV-I (MOI = 0.5) or left untreated. For bacterial infection, cells at 6 hpi were washed three times with PBS, fixed with 4% formaldehyde (RT, 1 h), permeabilized with 0.1% Triton X-100 (PBS, 10 min), blocked with 5% BSA (PBS, 1 h), incubated with anti-FLAG antibody (1:1000, overnight), and stained with Alexa Fluor 488-conjugated goat anti-mouse IgG (1:500, 2 h). For viral infection, cells at 18 hpi were washed three times with PBS, fixed with 4% paraformaldehyde, and processed similarly with anti-FLAG (1:1000) and anti-NS38 (1:5000) antibodies, followed by Alexa Fluor 488 (1:500) and Alexa Fluor 594 (1:500) conjugated secondary antibodies. Cells were counterstained with DAPI, washed with PBST, and imaged using a confocal microscope (SP8; Leica, Wetzlar, Germany).

To assess co-localization between gcHnf4α and caspase 3/caspase 9/AIF, CIK cells (3 × 10⁵ cells/well) were co-transfected with 400 ng GFP/gcHnf4α-GFP and 400 ng FLAG/caspase 3-FLAG/caspase 9-FLAG/AIF-FLAG. At 36 h post-transfection, cells were infected with *A. salmonicida* (MOI = 0.5) or left untreated. At 6 hpi, cells were rinsed with PBS and processed for immunofluorescence as described above.

To assess the impact of gcHnf4α on GCRV-I VIBs, CIK cells were seeded overnight in 24-well plates at 3 × 10^5^ cells/well, then transfected with 800 ng FLAG or gcHnf4α-FLAG. At 36 h post-transfection, cells were infected with GCRV-I (MOI = 0.5); after 1 h, the inoculum was removed, and cells were maintained in 2% FBS MEM at 28°C for persistent infection. At 18 hpi, cells were washed with PBS, and processed for immunofluorescence as described above. GCRV-I VIBs were quantified by analyzing average fluorescence intensity using Image J software.

### Bacterial infection assays *in vitro*

For antibacterial function assays, CIK cells (3 × 10⁵ cells/well) were transfected with 800 ng FLAG or gcHnf4α-FLAG for 36 h, then infected with *A. salmonicida* (MOI = 0.5). At 3 and 6 hpi, cells and supernatants were collected, serially diluted in PBS, and plated on BHI agar plates (ampicillin-containing) to quantify intracellular CFU by standard plate counting.

For caspase inhibition studies, CIK cells were transfected with FLAG/gcHnf4α-FLAG/gcHnf4α-LBD-FLAG/gcHnf4α-DBD-FLAG (800 ng) for 36 h, treated with 50 μM Z-DEVD-FMK/Z-LEHD-FMK (1 h), and infected with *A. salmonicida* (MOI = 0.5, 6 h). For AIF knockdown experiments, cells were co-transfected with FLAG/gcHnf4α constructs and 100 nM siRNA-control/siRNA-AIF, followed by bacterial infection and CFU enumeration.

For qRT-PCR analysis of apoptosis-related genes, FLAG/gcHnf4α-FLAG-transfected cells infected with *A. salmonicida* were collected at 6 hpi/24 hpi and processed for RNA extraction.

### Viral infection assays *in vitro*

For antiviral function assays, CIK cells (3 × 10⁵ cells/well) were transfected with 800 ng FLAG or gcHnf4α-FLAG for 36 h, then infected with GCRV-I (MOI = 0.02/0.2/2). At 18 hpi, supernatants were collected for viral titration (TCID₅₀ method), and cells were fixed with 4% PFA and stained with 1% crystal violet.

For caspase inhibition studies, cells were transfected with FLAG/gcHnf4α-FLAG (800 ng, 36 h), treated with 50 μM Z-DEVD-FMK/Z-LEHD-FMK (1 h), and infected with GCRV-I (MOI = 0.5). At 24 hpi, cells underwent crystal violet staining and viral gene expression analysis by qRT-PCR. For caspases 3/8/9 expression analysis, FLAG/gcHnf4α-FLAG-transfected cells infected with GCRV-I were collected at 24 hpi for qRT-PCR.

For analyses combining AIF knockdown with domain-specific constructs, CIK cells (3 × 10⁵ cells/well in 24-well plates) were seeded overnight, then co-transfected with 800 ng FLAG, gcHnf4α-FLAG, gcHnf4α-DBD-FLAG, or gcHnf4α-LBD-FLAG, plus 50 nM siRNA-AIF (Ribobio) or control siRNA as indicated. For experiments involving caspase inhibitors and domain-specific constructs, CIK cells (3 × 10⁵ cells/well in 24-well plates) were seeded overnight, transfected with 800 ng of the aforementioned FLAG-tagged constructs, and at 36 h post-transfection, pretreated with 50 μM Z-DEVD-FMK (Selleck #S7313), Z-LEHD-FMK (Selleck #S7312), or DMSO for 1 h. All cells were then infected with GCRV-I (MOI = 0.5) at 28°C for 1 h, after which viral inoculum was removed and cells were maintained in 2% FBS MEM at 28°C. Supernatants were collected at 18 hpi for viral titration via TCID₅₀ assay.

For caspase 3/9 activity assays, CIK cells (1 × 10⁶ cells/well in 6-well plates) were seeded overnight, co-transfected with 1000 ng target plasmid and 100 nM siRNA-control or siRNA-AIF, then infected with GCRV-I (MOI = 0.5) at 36 h post-transfection. At 18 hpi, cells and supernatants were combined, centrifuged (600 × g, 5 min, 4°C), and pellets were washed with PBS. Cells were lysed (100 μL lysis buffer per 2 × 10⁶ cells) on ice for 15 min, then centrifuged (21,000 × g, 15 min, 4°C). Supernatants were assayed for caspase activity using chromogenic substrates (Ac-DEVD-pNA for caspase 3, Ac-LEHD-pNA for caspase 9; Beyotime #C1116 and #C1158) at a final concentration of 200 mM. Caspase activity was quantified by measuring absorbance of hydrolyzed pNA at 405 nm, following the manufacturer’s protocol.

### Bacterial-viral coinfection assays *in vitro*

For coinfection assays, CIK cells (3 × 10⁵ cells/well) were transfected with 800 ng FLAG or gcHnf4α-FLAG for 36 h, washed with PBS, and infected with *A. salmonicida* (MOI = 0.5, 1.5 h). After replacing with 4% FBS MEM, cells were infected with GCRV-I (MOI = 0.5, 1 h), washed to remove unattached virus, and cultured in 2% FBS MEM. At 18 hpi/24 hpi, cells/supernatants were collected for CFU counting, crystal violet staining, and qRT-PCR analysis of viral genes and caspases 3/8/9.

For caspase inhibition in coinfection, cells were transfected with FLAG/gcHnf4α-FLAG (800 ng, 36 h), treated with 50 μM Z-DEVD-FMK/Z-LEHD-FMK (1 h), and subjected to coinfection as above. At 24 hpi, cells were analyzed by crystal violet staining and viral gene qRT-PCR.

### Cell death assays

Lactate dehydrogenase (LDH), a stable cytosolic enzyme released upon cell lysis, was measured using the CytoTox 96 Non-Radioactive Cytotoxicity Assay (Promega). CIK cells (1.5 × 10⁵ cells/well, 48-well plates) were transfected with 400 ng FLAG or gcHnf4α-FLAG for 36 h, infected with *A. salmonicida* (MOI = 0.5, 6 h), and supernatants collected after centrifugation (600 × g, 5 min). LDH activity was determined by adding 50 μL supernatant to 96-well plates, followed by equal volumes of CytoTox 96 reagent (30 min, RT, dark) and stop solution. Absorbance at 490 nm was measured using a microplate reader. Cell death percentage was calculated as: % Cell Death = [(A₄₉₀ (sample) – A₄₉₀ (uninfected))/ (A₄₉₀ (lysis control) – A₄₉₀ (uninfected))] × 100.

### Cell viability assays

Cell viability was assessed using the Cell Counting Kit-8 (CCK-8). CIK cells (1.5 × 10⁵ cells/well, 48-well plates) were transfected with 400 ng FLAG or gcHnf4α-FLAG, infected with *A. salmonicida*, GCRV-I, or coinfected, and treated with 50 μM Z-DEVD-FMK/Z-LEHD-FMK (1 h) where indicated. At specified time points (6, 24, 48 hpi for Fig 2C; 24 hpi for Figs 2G, 7D, 7H), culture supernatants were removed, cells washed with MEM, and 200 μL CCK-8 solution (MEM:CCK-8 = 100:1) added. After 4 h incubation (37°C), absorbance at 450 nm was measured to determine viability.

### Caspase activity assays

Caspase 3/8/9 activities were evaluated using chromogenic substrates. CIK cells (1 × 10⁶ cells/well, 6-well plates) were transfected with 2000 ng FLAG or gcHnf4α-FLAG for 36 h or co-transfected with 2000 ng FLAG or gcHnf4α-FLAG and 100 nM siRNA-control/siRNA-AIF for 36 h, and then infected with pathogens (MOI = 0.5, 6 h). The supernatants and cells were collected together and centrifuged at 4°C, 600 × g for 5 minutes. Supernatants (21,000 × g, 15 min, 4°C) were assayed for caspase activity using Ac-DEVD-pNA (caspase 3), Ac-IETD-pNA (caspase 8), or Ac-LEHD-pNA (caspase 9) according to the manufacturer’s protocol (Beyotime).

### Apoptosis assays

Flow cytometry was used to analyze apoptosis with an Annexin V-FITC/PI kit. CIK cells (1 × 10⁶ cells/well, 6-well plates) were transfected with 2000 ng FLAG/gcHnf4α-FLAG for 36 h, co-transfected with 2000 ng FLAG/gcHnf4α-FLAG and 100 nM siRNA-control/siRNA-AIF for 36 h, or treated with Z-DEVD-FMK/Z-LEHD-FMK (50 μM, 1 h), then infected with *A. salmonicida* (MOI = 0.5, 6 hpi). Cells were analyzed by flow cytometry as previously described [66].

### *In vivo* infection assays

Zebrafish larvae were microinjected at the 1–2-cell stage with FLAG or gcHnf4α-FLAG plasmids (200 ng/μL, 2 nL). At 4 dpf, larvae were infected by immersion with *A. salmonicida* (1 × 10⁷ CFU/mL), GCRV-II (2.76 × 10¹⁰ copies/mL), or coinfected. For caspase inhibition, larvae were treated with Z-DEVD-FMK/Z-LEHD-FMK (75 μM, 12 h) before infection. Rescue experiments in *hnf4α* ⁻ / ⁻ larvae (*hnf4α*^−/−^-2IS) involved microinjection of FLAG, caspase 3-FLAG, or caspase 9-FLAG plasmids. Larvae were maintained in sterile dishes with fresh water, and survival was monitored daily for 6 days (3 replicates/group, 20–30 larvae/replicate). Survival curves were generated using GraphPad Prism 9.0.

For pathogen interaction assays and analysis of *zfhnf4α*-mediated regulation of IFNs and inflammatory cytokines, 4 dpf *hnf4α*^−/−^-2IS and WT zebrafish larvae were allocated to four groups: uninfected controls, *A. salmonicida* infection (1 × 10⁷ cfu/mL), GCRV-II infection (2.76 × 10¹⁰ copies/mL), or mixed infection (both pathogens at the aforementioned concentrations). Larvae were exposed to 5 mL of culture water containing pathogens, followed by addition of 20 mL fresh water at 6 hpi. At 48 hpi, 10 larvae per group were processed for bacterial colony counting: washed twice with PBS, homogenized in 1 mL PBS with a sterile grinding rod, serially diluted, and plated on BHI agar with ampicillin. Plates were incubated at 28°C for 18 h before colony enumeration. Concurrent groups of 10 larvae were collected at 48 hpi for viral titer quantification and gene expression analysis via qRT-PCR.

### RNA extraction, reverse transcription, and qRT-PCR

Total RNA was extracted using TRIzol reagent, treated with RNase-free DNase I (37°C, 30 min), and reverse-transcribed with the RevertAid First Strand cDNA Synthesis Kit. qRT-PCR was performed on a QuantStudio 3 system (Thermo Fisher Scientific) with the following cycling conditions: 95°C for 3 min, followed by 50 cycles of 95°C for 15 s, 52–60°C for 15 s, and 72°C for 30 s. Primers were validated by sequencing and melting curve analysis. Target gene expression was normalized to the average of *β-actin*, *EF-1α*, and *18S* rRNA housekeeping genes and expressed as fold changes relative to controls. Primer sequences are listed in S2 Table.

### Co-Immunoprecipitation and Western Blotting

To investigate protein-protein interactions between gcHnf4α (or its truncated constructs) and caspase 3, caspase 9, or AIF, CIK cells (6 × 10⁶ cells/10 cm dish) were transfected with indicated plasmids (6 µg per plasmid) and infected with *A. salmonicida* (MOI = 0.5) at 36 h post-transfection. At 6 hpi, cells were lysed in IP buffer containing protease inhibitor cocktail (Thermo Fisher Scientific). For Co-IP, Anti-FLAG M2-Agarose Affinity Gel (#A2220, Sigma-Aldrich) was added to lysates and incubated overnight at 4°C with gentle rotation. Eluted proteins and input samples were analyzed by Western blotting using anti-FLAG (1:1000, #F3165, Sigma-Aldrich), anti-pTurbo-GFP (1:2000, #AB513, Evrogen), or anti-HA (1:1000, #51064–2-AP, Proteintech) antibodies.

To detect endogenous Hnf4α interactions in CIK cells, 40 μL Pierce Protein G Agarose (Thermo #UH284933) was washed twice with ice-cold PBS (1000 × g, 1 min, 4°C) in a 1.5 mL tube, then resuspended in 500 μL IP lysis buffer containing Protease Inhibitor Cocktail and 1 mg Hnf4α-Rabbit antibody. Following overnight incubation at 4°C with gentle mixing, beads were pelleted (1000 × g, 1 min, 4°C), washed twice with 1 mL pre-cooled PBS, and incubated with 600 μL protein sample overnight at 4°C. After five washes with PBS (2000 × g, 1 min, 4°C), bound proteins were eluted, and eluates/inputs analyzed via Western blotting using antibodies against GAPDH-Mouse (Proteintech #60004–1-Ig, 1:5000), Hnf4α-Rabbit (Dia-An #03319, 1:2000), or caspase 9-Rabbit (Proteintech #10380–1-AP, 1:500).

For exogenous zfHnf4α detection, WT zebrafish caudal fin cells were transfected with FLAG or zfHnf4α-FLAG (36 h), lysed, and probed with anti-FLAG (1:1000) or anti-GAPDH (1:5000, loading control). For exogenous gcHnf4α detection, CIK were transfected with FLAG or gcHnf4α-FLAG (36 h), lysed, and probed with anti-FLAG (1:1000) or anti-GAPDH (1:5000, loading control). For endogenous zfHnf4α analysis, 50 larvae (7 dpf) from WT and *hnf4α* ⁻ / ⁻ -2IS zebrafish were homogenized, and lysates were blotted with anti-Hnf4α (1:1000) or anti-GAPDH (1:5000).

### ChIP assay

Chromatin immunoprecipitation (ChIP) was performed using the SimpleChIP Plus Enzymatic Chromatin IP Kit (#9005, Cell Signaling Technology) to validate Hnf4α binding to AIF, Casp3, and Casp9 promoters. CIK cells (6 × 10⁶ cells/10 cm dish) were transfected with 12 µg YFP-FLAG (control) or gcHnf4α-FLAG, infected with *A. salmonicida* (MOI = 0.5), and maintained in 4% FBS MEM. At 6 hpi, chromatin was cross-linked with 1% formaldehyde (15 min), quenched with 125 mM glycine (5 min), and lysed in ChIP Buffer (#7008). Chromatin was digested with micrococcal nuclease (0.5 µL, 37°C, 5 min), fragmented by sonication, and centrifuged (9,400 g, 10 min, 4°C). Digested chromatin (150–900 bp) was subjected to immunoprecipitation with anti-FLAG antibody (1 µg) or Normal Rabbit IgG (negative control) overnight, followed by Protein G magnetic beads (#9006, 2 h, 4°C). After washing, DNA was de-crosslinked with 5 M NaCl and Proteinase K, purified, and amplified by PCR using promoter-specific primers. Amplicons were resolved by 2% agarose gel electrophoresis.

### Statistical analysis

Statistical analyses and graphs were generated using GraphPad Prism 9.0. qRT-PCR data are presented as mean ± standard error of the mean (SEM). Significance was determined by two-tailed Student’s t-test (*p < 0.05, **p < 0.01, ***p < 0.001) or one-way ANOVA. Survival curves were analyzed using the log-rank test.

## Supporting information

S1 Fig Amino acid sequence alignment of the DNA-binding domain (DBD; A) and ligand-binding domain (LBD; B) of Hnf4α across human, mouse, zebrafish, and grass carp. GenBank accession numbers of the sequences used are provided in S3 Table.
(TIF)

S2 Fig Phylogeny of vertebrate Hnf4α and specificity of anti-Hnf4α antibody. (A) Phylogenetic analysis of Hnf4α in vertebrates. GenBank accession numbers of the sequences used are provided in S3 Table. (B) Detection of endogenous and exogenous gcHnf4α in CIK cells using anti-Hnf4α or anti-FLAG antibody. (C) Detection of endogenous and exogenous zfHnf4α in zebrafish caudal fin cells using anti-Hnf4α or anti-FLAG antibody. Exogenously expressed gcHnf4α-FLAG and zfHnf4α-FLAG serve as molecular weight references for endogenous proteins.
(TIF)

S3 Fig Overexpression of gcHnf4α on the production of GCRV VIBs. (A and B) Immunofluorescence analysis for NS38 or NS80 in CIK cells transfected with FLAG or gcHnf4α-FLAG. Scale bars, 30 µM. (C and D) The average fluorescence intensity of NS38 or NS80 in CIK cells transfected with FLAG or gcHnf4α-FLAG. **p < 0.01.
(TIF)

S4 Fig gcHnf4α interacts with caspase 9 and mediates antiviral activity via AIF-dependent caspase activation. (A) gcHnf4α interacts with endogenous caspase 9. (B) In GCRV-infected cells, inhibition of caspase 3 or 9 abrogates gcHnf4α-mediated viral restriction. (C) AIF knockdown impairs gcHnf4α-induced activation of caspase 3 and 9 during GCRV infection. (D) AIF knockdown blocks gcHnf4α-mediated viral restriction in GCRV-infected cells. For all panels, data are mean ± SEM (n = 3). Statistical significance was determined by Student’s t-test (*p < 0.05, **p < 0.01; ns = not significant).
(TIF)

S5 Fig Promoter regions of *aif*, *caspase 3*, *caspase 9* and their putative HNF4A consensus motifs. (A-C) Promoter regions of *aif* (A), *caspase 3* (B) and *caspase 9* (C). (D) Putative HNF4A consensus motifs in the promoter regions of *aif*, *caspase 3* and *caspase 9*. (E) HNF4A consensus motif sequence (VAAABBKVM; V = A/C/G, B = T/C/G, K = T/G, M = A/C).
(TIF)

S6 Fig gcHnf4α exhibits selective binding to *caspase 3* promoter HNF4A motif. (A-B) ChIP-PCR analysis reveals gcHnf4α does not bind two putative HNF4A motifs in the *aif* promoter. (C-D) gcHnf4α specifically binds to *caspase 3* promoter Site 2 but not Site 1. (E) gcHnf4α fails to bind the putative HNF4A motif in the *caspase 9* promoter. CIK cells were transfected with YFP-FLAG or gcHnf4α-FLAG, left untreated or infected with *A. salmonicida* (MOI = 0.5), and harvested at 6 hpi for ChIP-PCR.
(TIF)

S7 Fig Obtaining *hnf4*α knockout zebrafish via Cas9/gRNA system. (A) Cartoon showing the position of the target site and its sequence in the *hnf4α* locus in zebrafish. (B) Representative Sanger sequencing results of the PCR amplicons from WT, *hnf4α*^+/-^ and *hnf4α*^-/-^-2IS zebrafish larvae with insertions of 2 base-pairs. (C) The endogenous zfHnf4α expression in WT and *hnf4α*^-/-^-2IS zebrafish larvae. Knockout of *hnf4α* was examined by analyzing zfHnf4α expression using immunoblotting with zfHnf4α polyclonal antibody in the WT and *hnf4α*^-/-^-2IS zebrafish larvae collected at 7 dpf.
(TIF)

S1 Table Comparison of the full-length, DBD and LBD sequences of gcHnf4α and zfHnf4α isoforms. (DOCX)

S2 Table The primer sequences. (DOCX)

S3 Table The GenBank accession numbers of Hnf4α sequences used for sequence alignment and phylogenetic tree analysis in the present study. (DOCX)

S1 Appendix Amino acid sequence alignment of gcHnf4α and zfHnf4α splicing isoforms. Accession numbers of zfHnf4α splicing isoforms are listed in S3 Table; the polypeptide sequence used for anti-Hnf4α antibody generation is highlighted in yellow.
(DOCX)

S1 Data File containing the original data used to build graphs. (XLSX)
