# Supplementary figures and images for: Hnf4α integrates AIF and caspase 3/9 signaling to restrict single and coinfecting pathogens in teleosts
Source: PLoS Pathog. 2025 Sep 8;21(9):e1013491. doi: 10.1371/journal.ppat.1013491 (PMC12425335; doi:10.1371/journal.ppat.1013491)

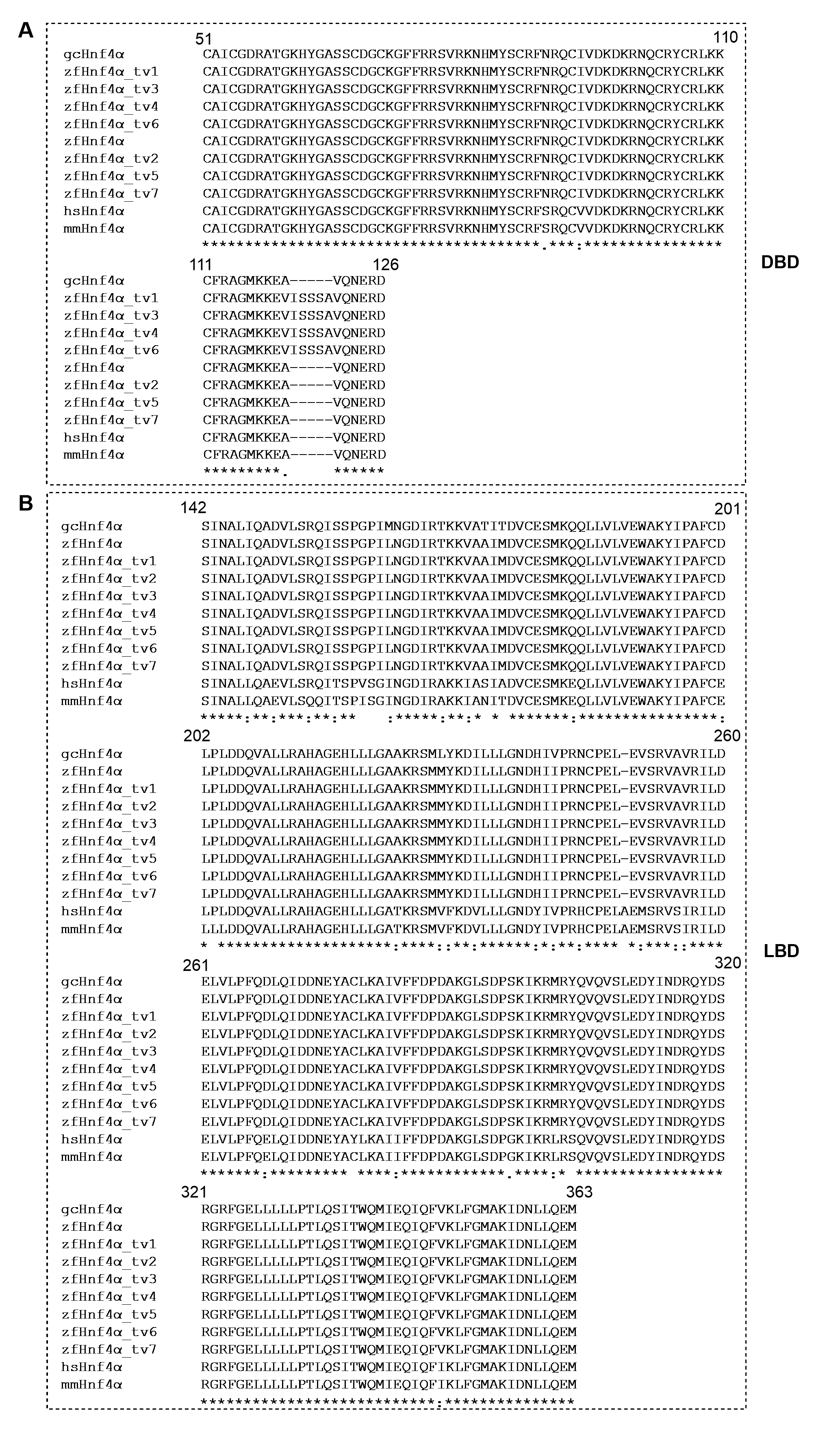

Supplement: S1 Fig — GenBank accession numbers of the sequences used are provided in S3 Table. (TIF) [file ppat.1013491.s001.tif]

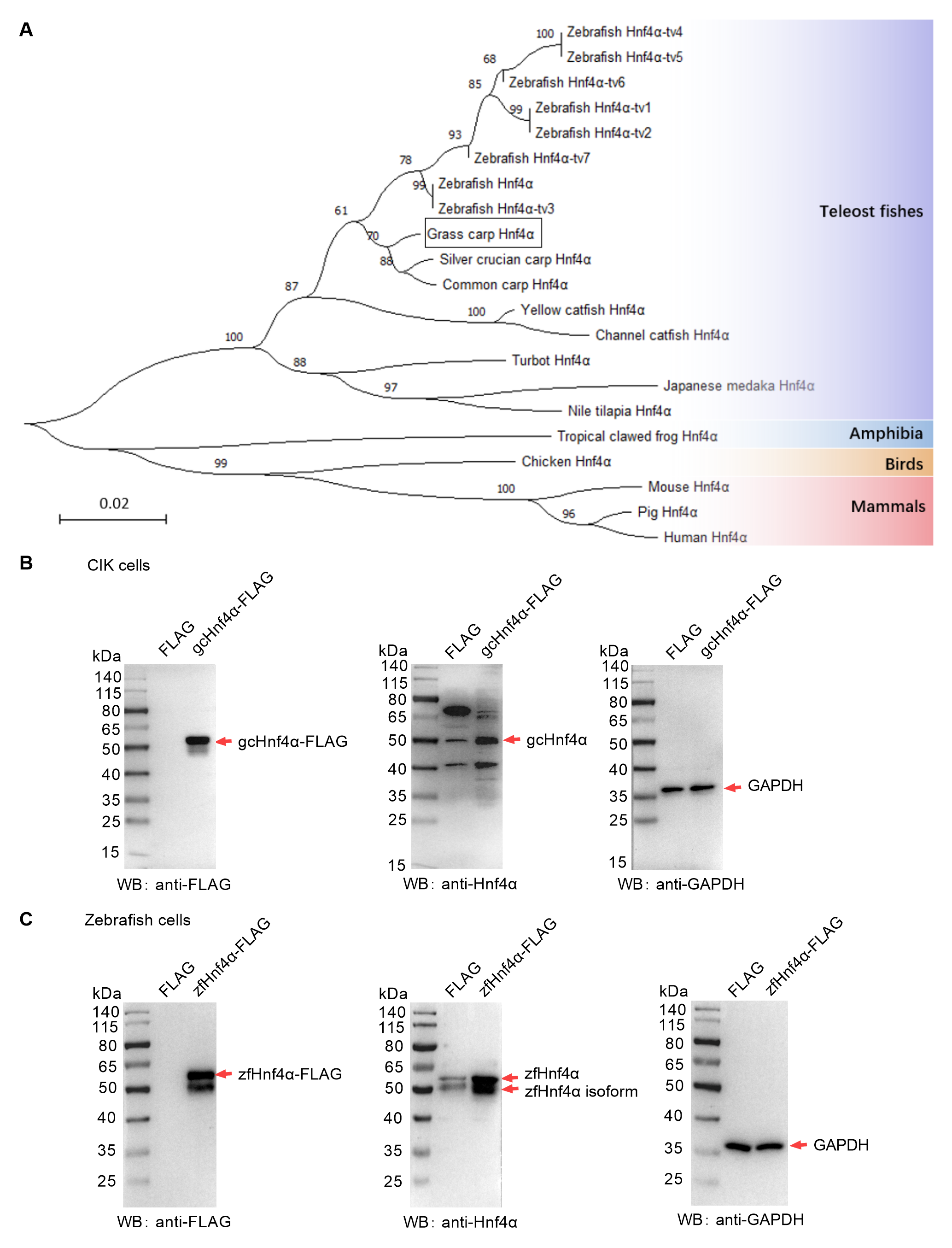

Supplement: S2 Fig — (A) Phylogenetic analysis of Hnf4α in vertebrates. GenBank accession numbers of the sequences used are provided in S3 Table. (B) Detection of endogenous and exogenous gcHnf4α in CIK cells using anti-Hnf4α or anti-FLAG antibody. (C) Detection of endogenous and exogenous zfHnf4α in zebrafish caudal fin cells using anti-Hnf4α or anti-FLAG antibody. Exogenously expressed gcHnf4α-FLAG and zfHnf4α-FLAG serve as molecular weight references for endogenous proteins. (TIF) [file ppat.1013491.s002.tif]

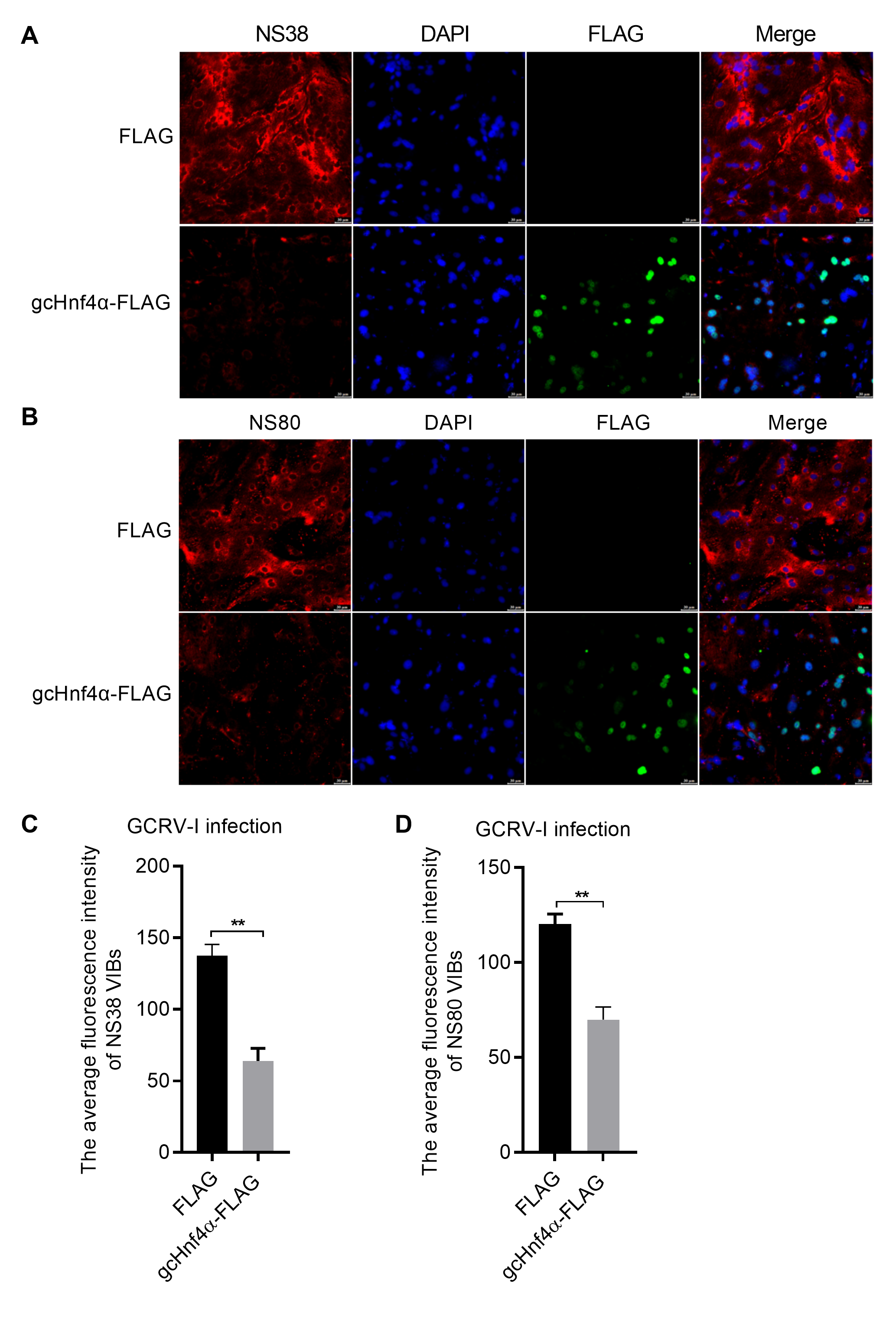

Supplement: S3 Fig — (A and B) Immunofluorescence analysis for NS38 or NS80 in CIK cells transfected with FLAG or gcHnf4α-FLAG. Scale bars, 30 µM. (C and D) The average fluorescence intensity of NS38 or NS80 in CIK cells transfected with FLAG or gcHnf4α-FLAG. **p < 0.01. (TIF) [file ppat.1013491.s003.tif]

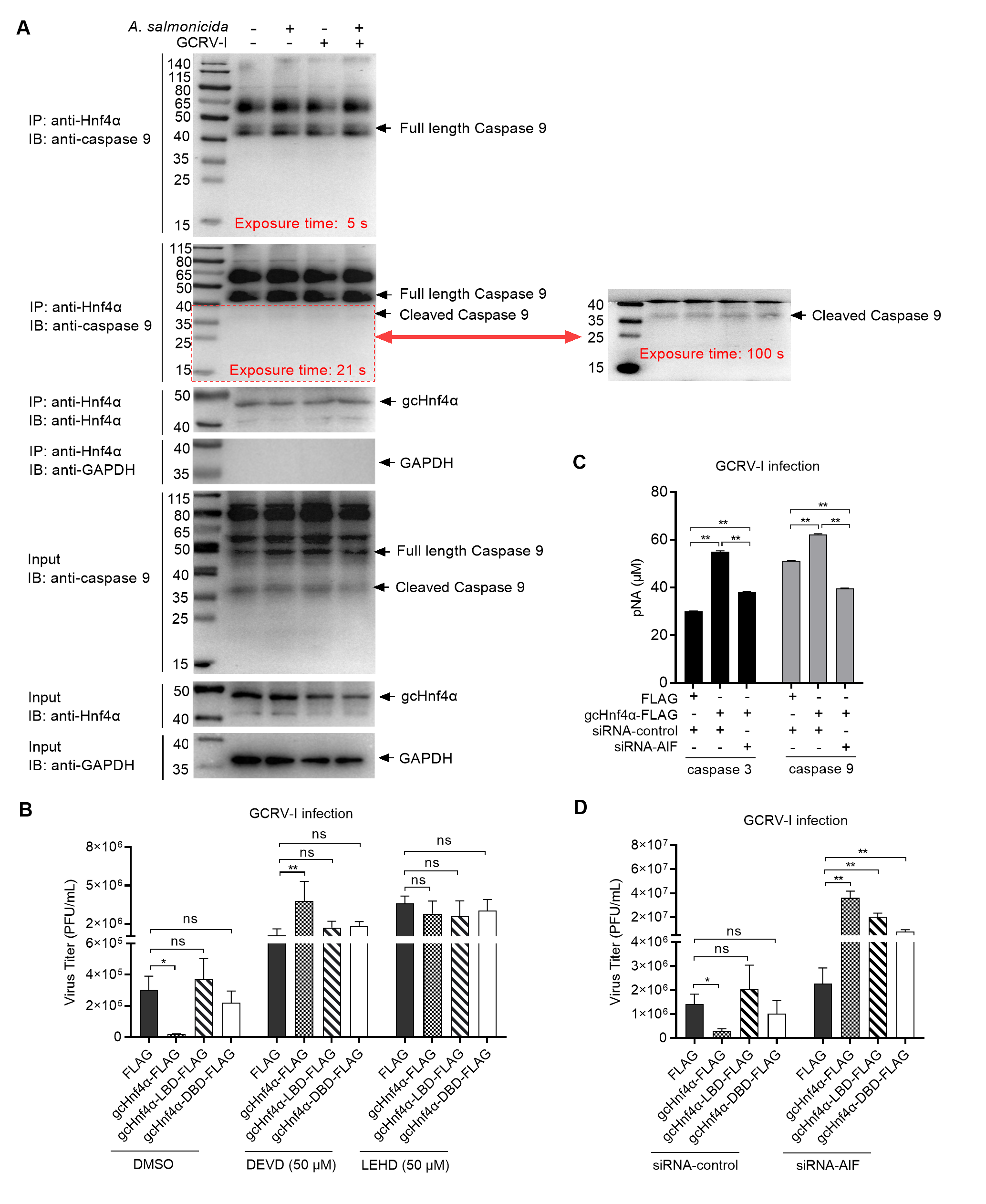

Supplement: S4 Fig — (A) gcHnf4α interacts with endogenous caspase 9. (B) In GCRV-infected cells, inhibition of caspase 3 or 9 abrogates gcHnf4α-mediated viral restriction. (C) AIF knockdown impairs gcHnf4α-induced activation of caspase 3 and 9 during GCRV infection. (D) AIF knockdown blocks gcHnf4α-mediated viral restriction in GCRV-infected cells. For all panels, data are mean ± SEM (n = 3). Statistical significance was determined by Student’s t-test (*p < 0.05, **p < 0.01; ns = not significant). (TIF) [file ppat.1013491.s004.tif]

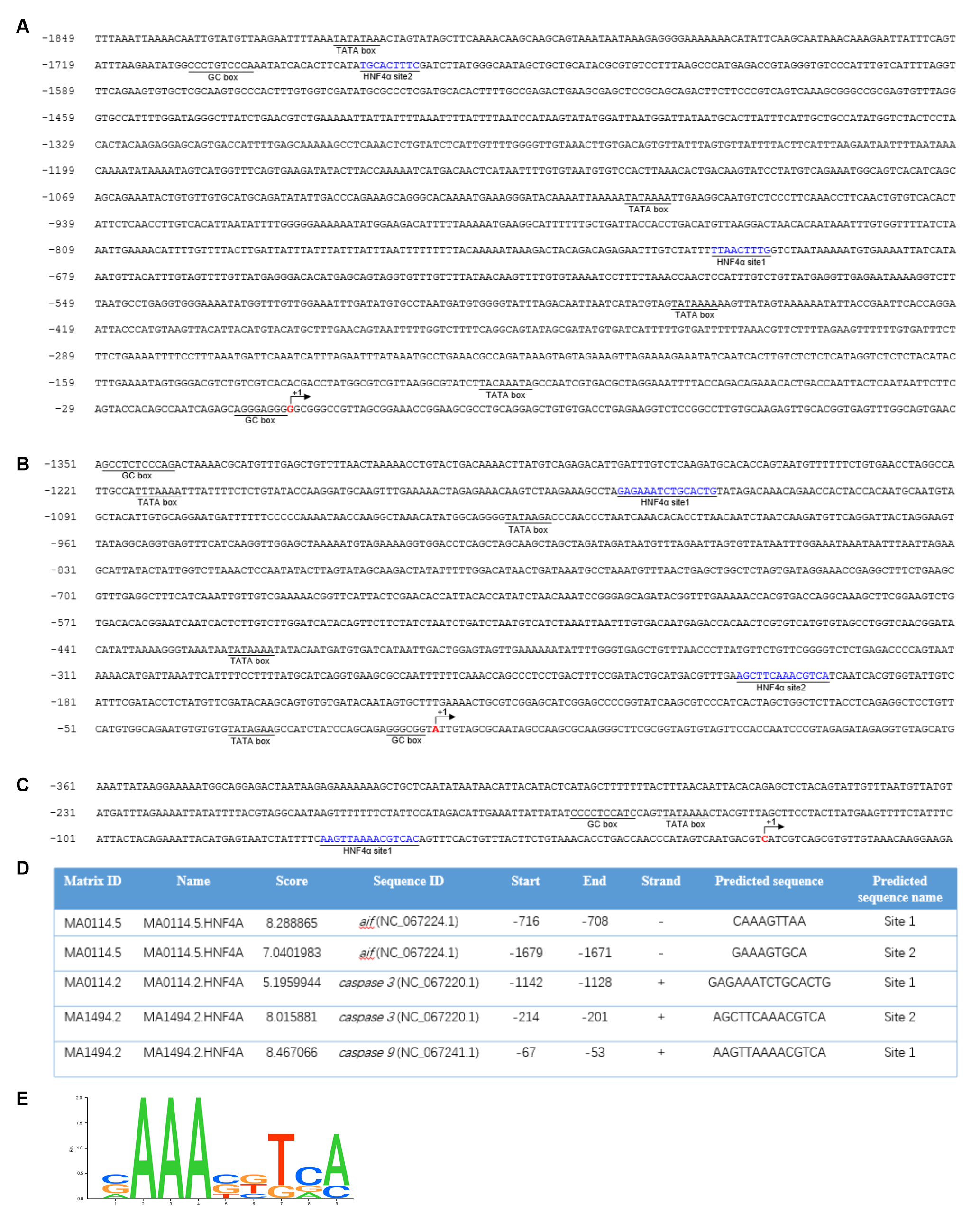

Supplement: S5 Fig — (A-C) Promoter regions of aif (A), caspase 3 (B) and caspase 9 (C). (D) Putative HNF4A consensus motifs in the promoter regions of aif, caspase 3 and caspase 9. (E) HNF4A consensus motif sequence (VAAABBKVM; V = A/C/G, B = T/C/G, K = T/G, M = A/C). (TIF) [file ppat.1013491.s005.tif]

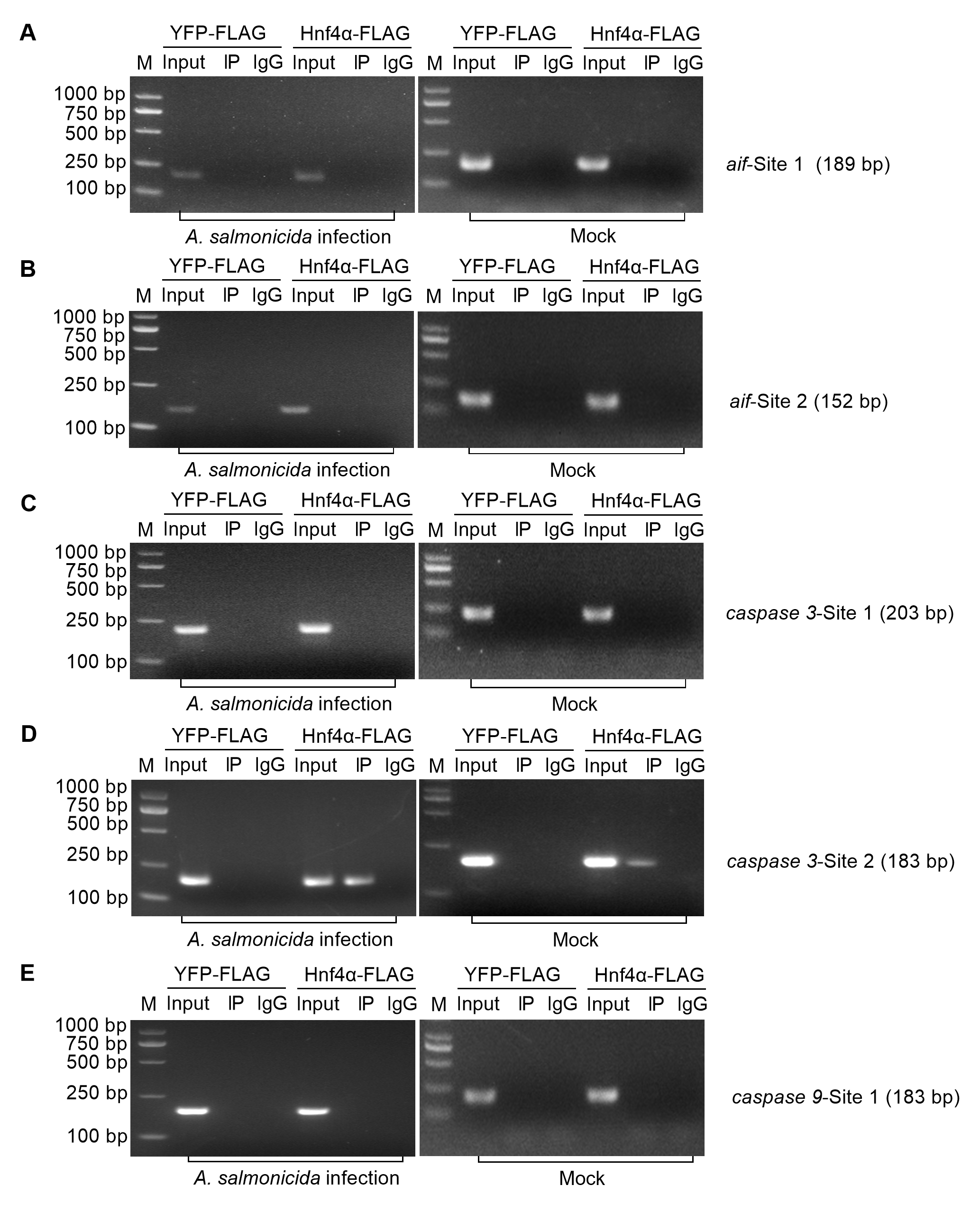

Supplement: S6 Fig — (A-B) ChIP-PCR analysis reveals gcHnf4α does not bind two putative HNF4A motifs in the aif promoter. (C-D) gcHnf4α specifically binds to caspase 3 promoter Site 2 but not Site 1. (E) gcHnf4α fails to bind the putative HNF4A motif in the caspase 9 promoter. CIK cells were transfected with YFP-FLAG or gcHnf4α-FLAG, left untreated or infected with A. salmonicida (MOI = 0.5), and harvested at 6 hpi for ChIP-PCR. (TIF) [file ppat.1013491.s006.tif]

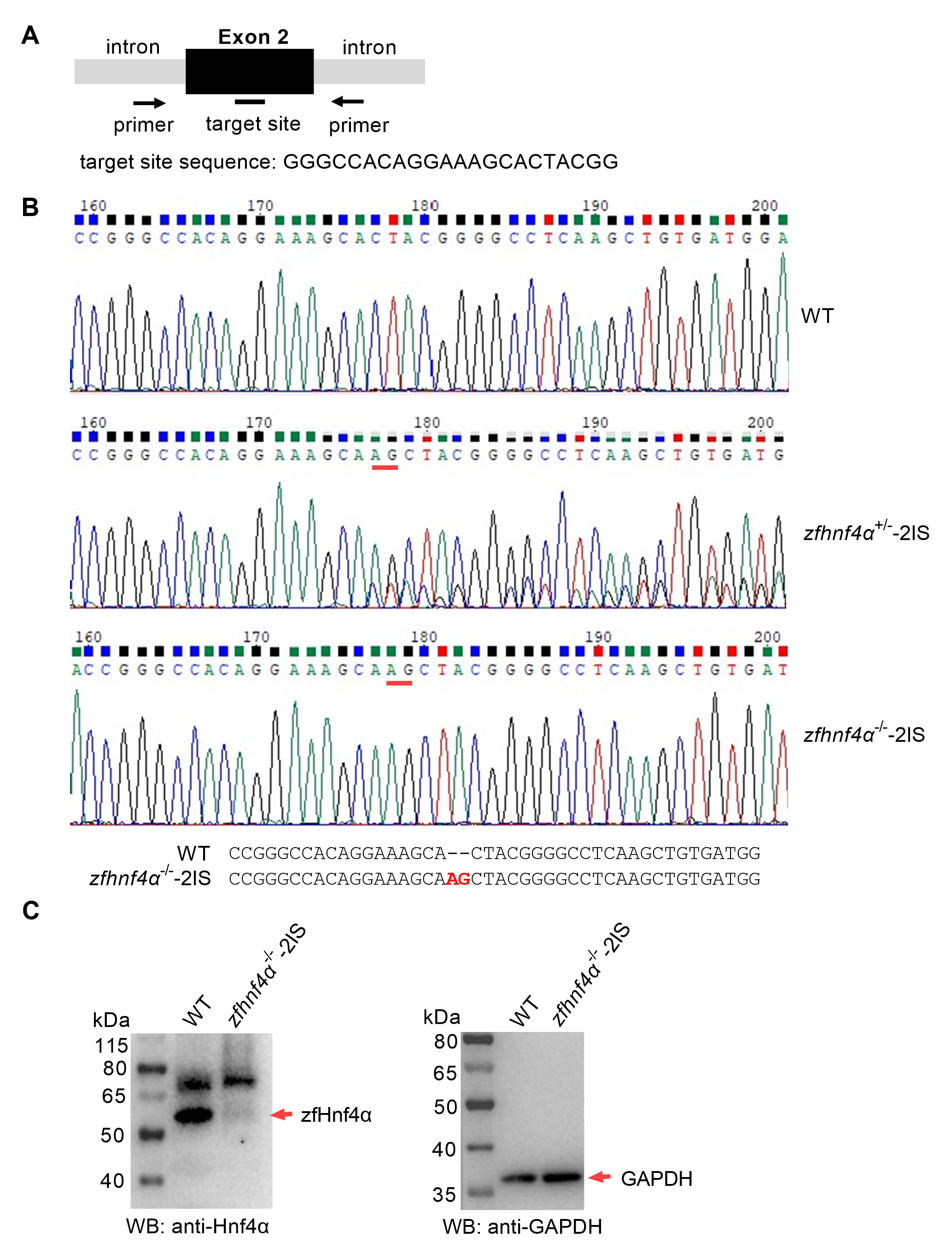

Supplement: S7 Fig — (A) Cartoon showing the position of the target site and its sequence in the hnf4α locus in zebrafish. (B) Representative Sanger sequencing results of the PCR amplicons from WT, hnf4α+/- and hnf4α-/--2IS zebrafish larvae with insertions of 2 base-pairs. (C) The endogenous zfHnf4α expression in WT and hnf4α-/--2IS zebrafish larvae. Knockout of hnf4α was examined by analyzing zfHnf4α expression using immunoblotting with zfHnf4α polyclonal antibody in the WT and hnf4α-/--2IS zebrafish larvae collected at 7 dpf. (TIF) [file ppat.1013491.s007.tif]
