# Supplementary material for: Hnf4α integrates AIF and caspase 3/9 signaling to restrict single and coinfecting pathogens in teleosts
Source: PLoS Pathog. 2025 Sep 8;21(9):e1013491. doi: 10.1371/journal.ppat.1013491 (PMC12425335; doi:10.1371/journal.ppat.1013491)
Supplement: S1 Table — (DOCX) [file ppat.1013491.s008.docx]

**S1 Table. Comparison of the full-length, DBD and LBD sequences of gcHnf4α and zfHnf4α isoforms.** GenBank accession numbers of the sequences used are provided in S3 Table.

|  | gcHnf4α (full length) | gcHnf4α (DBD) | gcHnf4α (LBD) |
| --- | --- | --- | --- |
| zfHnf4α | 97.58 | 100 | 97.75 |
| zfHnf4α_tv1 | 90.43 | 93.83 | 97.75 |
| zfHnf4α_tv2 | 91.43 | 100 | 97.75 |
| zfHnf4α_tv3 | 94.66 | 93.83 | 97.75 |
| zfHnf4α_tv4 | 90.28 | 93.83 | 97.75 |
| zfHnf4α_tv5 | 91.27 | 100 | 97.75 |
| zfHnf4α_tv6 | 90.41 | 93.83 | 97.75 |
| zfHnf4α_tv7 | 91.41 | 100 | 97.75 |
