# Supplementary material for: Hnf4α integrates AIF and caspase 3/9 signaling to restrict single and coinfecting pathogens in teleosts
Source: PLoS Pathog. 2025 Sep 8;21(9):e1013491. doi: 10.1371/journal.ppat.1013491 (PMC12425335; doi:10.1371/journal.ppat.1013491)
Supplement: S2 Table — (DOCX) [file ppat.1013491.s009.docx]

**S2 Table. The primer sequences.**

| Primers | Sequences（5’-3’） | Application |
| --- | --- | --- |
| gcHnf4α-F | CGCAAGCTTATGGAGATGGCAGACTATA | Ligated to p3xFLAG-CMV™-14 vector |
| gcHnf4α-R | CGCGGATCCGATGGCCTCTTGTTTAGTG |  |
| zfHnf4α-F | CGCAAGCTTATGGAGATGGCAGACTATA |  |
| zfHnf4α-R | CGGGGTACCGAGATGGCCTCTTGTTTAGT |  |
| gcHnf4α-LBD-F | CCGGAATTCAATGTGTGCCATCTGTG |  |
| gcHnf4α-LBD-R | CGCGGATCCGTCTCTTTCATTCTGC |  |
| gcHnf4α-DBD-F | CCGGAATTCA ATGTCCATAAATGCAC |  |
| gcHnf4α-DBD-R | CGCGGATCCCATCTCTTGTAGCAGG |  |
| caspase 3-F | CTTGCGGCCGATGAACGGAGACTGCG |  |
| caspase 3-R | GCTCTAGAACCAGTGAAGTACATC |  |
| caspase 9-F | CCGGAATTCA ATGGTCCCTAATCACA |  |
| caspase 9-R | CGCGGATCCCAAAGACTGAAAGTAG |  |
| AIF-F | CCGGAATTCAATGTTCAAGTGCAAGACAG |  |
| AIF-R | CGCGGATCCGTCTTCGTGAATGTTG |  |
| gcHnf4α-F1 | CCGCTCGAGATGCGTTTGTCCAAACCAC | Ligated to pTurboGFP-N vector |
| gcHnf4α-R1 | GCGGATCCAAGATGGCCTCTTGTTTAGTG |  |
| caspase 3-F1 | CTCAGATCTATGAACGGAGACTGCG |  |
| caspase 3-R1 | CGGAATTCGACCAGTGAAGTACATC |  |
| AIF-F1 | CCGCTCGAGATGTTCAAGTGCAAGACAG |  |
| AIF-R1 | GCGGATCCACGTCTTCGTGAATGTTG |  |
| caspase 9-F1 | CGAAGCTTATGGTCCCTAATCACAGAC | Ligated to pcDNA3.1- FLAG-HA vector |
| caspase 9-R1 | CGTCTAGACAAAGACTGAAAGTAGAGATG |  |
| *β-actin*-F | GGCTGTGCTGTCCCTGTA | qRT-PCR for CIK cells |
| *β-actin*-R | GGCTGTGCTGTCCCTGTA |  |
| *EF1a*-F | CAGCACAAACATGGGCTGGTTC |  |
| *EF1a*-R | ACGGGTACAGTTCCAATACCTCCA |  |
| *18S*-F | ATTTCCGACACGGAGAGG |  |
| *18S*-R | CATGGGTTTAGGATACGCTC |  |
| NS38-F | CTATGGCACTGGCGTTTA |  |
| NS38-R | GTCGGGTAGTTCAGAGGG |  |
| NS80-F | GGAAGCCGACAAGGGAATG |  |
| NS80-R | TGGAGTAGCCGTGGGAAG |  |
| *gchnf4α*-F | GGTCCAGGAGCATCTCAGTA |  |
| *gchnf4α*-R | TGATGGTAGGTTGAGGGATG |  |
| *aif*-F | GATGGACGACTGGTGAAGAC |  |
| *aif*-R | TGATCATGATGCTCCACACG |  |
| *caspase 3*-F | CTGATGGGGCATCTGGACTG |  |
| *caspase 3*-R | GTTGGTTCATGCCTGTCGTG |  |
| *caspase 8*-F | GACTAGAAGAGCAAGCACTG |  |
| *caspase 8*-R | TGTACTCGGAGACACCTTTA |  |
| *caspase 9*-F | GGGATAGATGACCAGATGGA |  |
| *caspase 9*-R | TGTCCCTCCAAGAGACATAG |  |
| *bax*-F | ACGACAGGGATGGGTGATAC |  |
| *bax*-R | CGCAATGTCCCCTTATGTGG |  |
| *bcl-2*-F | TGCCTGTCTCTCCTCTTCAG |  |
| *bcl-2*-R | CAGAGAGGGTGAAAGAGCGT |  |
| *bcl-xl*-F | CTGTGAAAACGGTGATGGCA |  |
| *bcl-xl*-R | CAAACGTGCATGCCTATCCA |  |
| GCRV-II-S6-F | AGCGCAGCAGGCAATTACTATCT |  |
| GCRV-II-S6-R | ATCTGCTGGTAATGCGGAACG |  |
| *zfaif*-F | TCTGCTGACCCTGGTTTAGA | qRT-PCR for zebrafish larvae |
| *zfaif*-R | TACACCCTCTTTCCGGACTT |  |
| *zfcaspase 3*-F | TGTTCTTTATTCAGGCTTGTCG |  |
| *zfcaspase 3*-R | CTGCCATACTTTGTCATCATTT |  |
| *zfcaspase 9*-F | AGGCATTGAATCCCGAAGA |  |
| *zfcaspase 9*-R | ACAGGAGGGCGATGAACAC |  |
| *zfifn1*-F | GTCAGGACTAAAAACTTCAC |  |
| *zfifn1*-R | TCTTAATACACGCAAAGATGAGAACT |  |
| *zfifn3*-F | TTCTGCTTTGTGCAGGTTTG |  |
| *zfifn3*-R | GGTATAGAAACGCGGTCGTC |  |
| *zfil-8*-F | GTCGCTGCATTGAAACAGAA |  |
| *zfil-8*-R | CTTAACCCATGGAGCAGAGG |  |
| *zfil-1β*-F | GGCTGTGTGTTTGGGAATCT |  |
| *zfil-1β*-R | TGATAAACCAACCGGGACA |  |
| *zfil-17*-F | TGCCGACTGTCATACTGCG |  |
| *zfil-17*-R | TCTTTTTCCAACAATGGCACAC |  |
| *zftnfα*-F | GCGCTTTTCTGAATCCTACG |  |
| *zftnfα*-R | TGCCCAGTCTGTCTCCTTCT |  |
| *gapdh*-F | GATACACGGAGCACCAGGTT |  |
| *gapdh*-R | GCCATCAGGTCACATACACG |  |
| ch-AIF-s1-F | AAAGACTACAGACAGAGAATTTGT | ChIP-PCR |
| ch-AIF-s1-R | TTATTCTCAACCTCATAACAGAC |  |
| ch-AIF-s2-F | GCTTCAAAACAAGCAAGCAGTAA |  |
| ch-AIF-s2-R | TGCAGCAGCTATTGCCCATAA |  |
| ch-caspase 3-s1-F | AACCTAGGCCATTGCCATTTA |  |
| ch-caspase 3-s1-R | CCCCTGCCATATGTTTAGCCTT |  |
| ch-caspase 3-s2-F | CCTCCTGACTTTCCGATACTG |  |
| ch-caspase 3-s2-R | GAGGTAAGAGCCAGCTAGTGATG |  |
| ch-caspase 9-s1-F | CCATAGACATTGAAATTATTATATCC |  |
| ch-caspase 9-s1-R | ACGTCATTGACTATGGGTTGGTCAGG |  |
