# Supplementary material for: Hnf4α integrates AIF and caspase 3/9 signaling to restrict single and coinfecting pathogens in teleosts
Source: PLoS Pathog. 2025 Sep 8;21(9):e1013491. doi: 10.1371/journal.ppat.1013491 (PMC12425335; doi:10.1371/journal.ppat.1013491)
Supplement: S3 Table — (DOCX) [file ppat.1013491.s010.docx]

**S3 Table. The GenBank accession numbers of Hnf4α sequences used for sequence alignment and phylogenetic tree analysis in the present study.**

| Gene | Accession number | Gene | Accession number |
| --- | --- | --- | --- |
| zfHnf4α | NP_919349 | zfHnf4α_tv1 | XP_073794164 |
| zfHnf4α_tv2 | XP_073794165 | zfHnf4α_tv3 | XP_021325423 |
| zfHnf4α_tv4 | XP_021325424 | zfHnf4α_tv5 | XP_073794166 |
| zfHnf4α_tv6 | XP_068072779 | zfHnf4α_tv7 | XP_009295086 |
| Silver crucian carp Hnf4α | XP_052451031 | Yellow catfish Hnf4α | XP_027000374 |
| Common carp Hnf4α | XP_018926973 | Channel catfish Hnf4α | NP_001187505 |
| Turbot Hnf4α | XP_035501910 | Japanese medaka Hnf4α | XP_023812685 |
| Nile tilapia Hnf4α | XP_005477971 | Tropical clawed frog Hnf4α | XP_004918605 |
| Chicken Hnf4α | NP_001026026 | Pig Hnf4α | NP_001038036 |
| Mouse Hnf4α | XP_006498850 | Human Hnf4α | XP_054179379 |
