# Supplementary material for: Hnf4α integrates AIF and caspase 3/9 signaling to restrict single and coinfecting pathogens in teleosts
Source: PLoS Pathog. 2025 Sep 8;21(9):e1013491. doi: 10.1371/journal.ppat.1013491 (PMC12425335; doi:10.1371/journal.ppat.1013491)
Supplement: S1 Appendix — Accession numbers of zfHnf4α splicing isoforms are listed in S3 Table; the polypeptide sequence used for anti-Hnf4α antibody generation is highlighted in yellow. (DOCX) [file ppat.1013491.s011.docx]

**S1 Appendix**: Amino acid sequence alignment of gcHnf4α and zfHnf4α splicing isoforms. Accession numbers of zfHnf4α splicing isoforms are listed in S3 Table; the polypeptide sequence used for anti-Hnf4α antibody generation is highlighted in yellow.

gcHnf4α ---------MEMADYSEALDPAYTTLEFENMQVLAMSTDSSPAESANMNAANHLGAGTLC 51

zfHnf4α_tv4 ----------------------MVNVNAQVSTHMEAPYDSSPQESANMNAANHLGAGTLC 38

zfHnf4α_tv3 MRLSKPLVDMEMADYSEALDPAYTTLEFENMQVLAMSTDSSPQESANMNAANHLGAGTLC 60

zfHnf4α_tv1 ----------------------------MYEGHFSTTPDSSPQESANMNAANHLGAGTLC 32

zfHnf4α_tv6 ------------------------------------MNDSSPQESANMNAANHLGAGTLC 24

zfHnf4α_tv5 ----------------------MVNVNAQVSTHMEAPYDSSPQESANMNAANHLGAGTLC 38

zfHnf4α_tv2 ----------------------------MYEGHFSTTPDSSPQESANMNAANHLGAGTLC 32

zfHnf4α ---------MEMADYSEALDPAYTTLEFENMQVLAMSTDSSPQESANMNAANHLGAGTLC 51

zfHnf4α_tv7 ------------------------------------MNDSSPQESANMNAANHLGAGTLC 24

**** *****************

**DBD**

gcHnf4α AICGDRATGKHYGASSCDGCKGFFRRSVRKNHMYSCRFNRQCIVDKDKRNQCRYCRLKKC 111

zfHnf4α_tv4 AICGDRATGKHYGASSCDGCKGFFRRSVRKNHMYSCRFNRQCIVDKDKRNQCRYCRLKKC 98

zfHnf4α_tv3 AICGDRATGKHYGASSCDGCKGFFRRSVRKNHMYSCRFNRQCIVDKDKRNQCRYCRLKKC 120

zfHnf4α_tv1 AICGDRATGKHYGASSCDGCKGFFRRSVRKNHMYSCRFNRQCIVDKDKRNQCRYCRLKKC 92

zfHnf4α_tv6 AICGDRATGKHYGASSCDGCKGFFRRSVRKNHMYSCRFNRQCIVDKDKRNQCRYCRLKKC 84

zfHnf4α_tv5 AICGDRATGKHYGASSCDGCKGFFRRSVRKNHMYSCRFNRQCIVDKDKRNQCRYCRLKKC 98

zfHnf4α_tv2 AICGDRATGKHYGASSCDGCKGFFRRSVRKNHMYSCRFNRQCIVDKDKRNQCRYCRLKKC 92

zfHnf4α AICGDRATGKHYGASSCDGCKGFFRRSVRKNHMYSCRFNRQCIVDKDKRNQCRYCRLKKC 111

zfHnf4α_tv7 AICGDRATGKHYGASSCDGCKGFFRRSVRKNHMYSCRFNRQCIVDKDKRNQCRYCRLKKC 84

************************************************************

**DBD**

**LBD**

gcHnf4α FRAGMKKE-----AVQNERDRISTRRSSYEDSSLPSINALIQADVLSRQISSPGPIMNGD 166

zfHnf4α_tv4 FRAGMKKEVISSSAVQNERDRISTRRSSYEDSSLPSINALIQADVLSRQISSPGPILNGD 158

zfHnf4α_tv3 FRAGMKKEVISSSAVQNERDRISTRRSSYEDSSLPSINALIQADVLSRQISSPGPILNGD 180

zfHnf4α_tv1 FRAGMKKEVISSSAVQNERDRISTRRSSYEDSSLPSINALIQADVLSRQISSPGPILNGD 152

zfHnf4α_tv6 FRAGMKKEVISSSAVQNERDRISTRRSSYEDSSLPSINALIQADVLSRQISSPGPILNGD 144

zfHnf4α_tv5 FRAGMKKE-----AVQNERDRISTRRSSYEDSSLPSINALIQADVLSRQISSPGPILNGD 153

zfHnf4α_tv2 FRAGMKKE-----AVQNERDRISTRRSSYEDSSLPSINALIQADVLSRQISSPGPILNGD 147

zfHnf4α FRAGMKKE-----AVQNERDRISTRRSSYEDSSLPSINALIQADVLSRQISSPGPILNGD 166

zfHnf4α_tv7 FRAGMKKE-----AVQNERDRISTRRSSYEDSSLPSINALIQADVLSRQISSPGPILNGD 139

******** *******************************************:***

**LBD**

gcHnf4α IRTKKVATITDVCESMKQQLLVLVEWAKYIPAFCDLPLDDQVALLRAHAGEHLLLGAAKR 226

zfHnf4α_tv4 IRTKKVAAIMDVCESMKQQLLVLVEWAKYIPAFCDLPLDDQVALLRAHAGEHLLLGAAKR 218

zfHnf4α_tv3 IRTKKVAAIMDVCESMKQQLLVLVEWAKYIPAFCDLPLDDQVALLRAHAGEHLLLGAAKR 240

zfHnf4α_tv1 IRTKKVAAIMDVCESMKQQLLVLVEWAKYIPAFCDLPLDDQVALLRAHAGEHLLLGAAKR 212

zfHnf4α_tv6 IRTKKVAAIMDVCESMKQQLLVLVEWAKYIPAFCDLPLDDQVALLRAHAGEHLLLGAAKR 204

zfHnf4α_tv5 IRTKKVAAIMDVCESMKQQLLVLVEWAKYIPAFCDLPLDDQVALLRAHAGEHLLLGAAKR 213

zfHnf4α_tv2 IRTKKVAAIMDVCESMKQQLLVLVEWAKYIPAFCDLPLDDQVALLRAHAGEHLLLGAAKR 207

zfHnf4α IRTKKVAAIMDVCESMKQQLLVLVEWAKYIPAFCDLPLDDQVALLRAHAGEHLLLGAAKR 226

zfHnf4α_tv7 IRTKKVAAIMDVCESMKQQLLVLVEWAKYIPAFCDLPLDDQVALLRAHAGEHLLLGAAKR 199

*******:* **************************************************

**LBD**

gcHnf4α SMLYKDILLLGNDHIVPRNCPELEVSRVAVRILDELVLPFQDLQIDDNEYACLKAIVFFD 286

zfHnf4α_tv4 SMMYKDILLLGNDHIIPRNCPELEVSRVAVRILDELVLPFQDLQIDDNEYACLKAIVFFD 278

zfHnf4α_tv3 SMMYKDILLLGNDHIIPRNCPELEVSRVAVRILDELVLPFQDLQIDDNEYACLKAIVFFD 300

zfHnf4α_tv1 SMMYKDILLLGNDHIIPRNCPELEVSRVAVRILDELVLPFQDLQIDDNEYACLKAIVFFD 272

zfHnf4α_tv6 SMMYKDILLLGNDHIIPRNCPELEVSRVAVRILDELVLPFQDLQIDDNEYACLKAIVFFD 264

zfHnf4α_tv5 SMMYKDILLLGNDHIIPRNCPELEVSRVAVRILDELVLPFQDLQIDDNEYACLKAIVFFD 273

zfHnf4α_tv2 SMMYKDILLLGNDHIIPRNCPELEVSRVAVRILDELVLPFQDLQIDDNEYACLKAIVFFD 267

zfHnf4α SMMYKDILLLGNDHIIPRNCPELEVSRVAVRILDELVLPFQDLQIDDNEYACLKAIVFFD 286

zfHnf4α_tv7 SMMYKDILLLGNDHIIPRNCPELEVSRVAVRILDELVLPFQDLQIDDNEYACLKAIVFFD 259

**:************:********************************************

**LBD**

gcHnf4α PDAKGLSDPSKIKRMRYQVQVSLEDYINDRQYDSRGRFGELLLLLPTLQSITWQMIEQIQ 346

zfHnf4α_tv4 PDAKGLSDPSKIKRMRYQVQVSLEDYINDRQYDSRGRFGELLLLLPTLQSITWQMIEQIQ 338

zfHnf4α_tv3 PDAKGLSDPSKIKRMRYQVQVSLEDYINDRQYDSRGRFGELLLLLPTLQSITWQMIEQIQ 360

zfHnf4α_tv1 PDAKGLSDPSKIKRMRYQVQVSLEDYINDRQYDSRGRFGELLLLLPTLQSITWQMIEQIQ 332

zfHnf4α_tv6 PDAKGLSDPSKIKRMRYQVQVSLEDYINDRQYDSRGRFGELLLLLPTLQSITWQMIEQIQ 324

zfHnf4α_tv5 PDAKGLSDPSKIKRMRYQVQVSLEDYINDRQYDSRGRFGELLLLLPTLQSITWQMIEQIQ 333

zfHnf4α_tv2 PDAKGLSDPSKIKRMRYQVQVSLEDYINDRQYDSRGRFGELLLLLPTLQSITWQMIEQIQ 327

zfHnf4α PDAKGLSDPSKIKRMRYQVQVSLEDYINDRQYDSRGRFGELLLLLPTLQSITWQMIEQIQ 346

zfHnf4α_tv7 PDAKGLSDPSKIKRMRYQVQVSLEDYINDRQYDSRGRFGELLLLLPTLQSITWQMIEQIQ 319

************************************************************

**LBD**

gcHnf4α FVKLFGMAKIDNLLQEMLLGGSANEAPHSHHSLHPHLVQEHLSNNVIVTTNMATPIHNGQ 406

zfHnf4α_tv4 FVKLFGMAKIDNLLQEMLLGGSANEAPHAHHSLHPHLVQEHLSNNVIVTANMATPLHNGQ 398

zfHnf4α_tv3 FVKLFGMAKIDNLLQEMLLGGSANEAPHAHHSLHPHLVQEHLSNNVIVTANMATPLHNGQ 420

zfHnf4α_tv1 FVKLFGMAKIDNLLQEMLLGGSANEAPHAHHSLHPHLVQEHLSNNVIVTANMATPLHNGQ 392

zfHnf4α_tv6 FVKLFGMAKIDNLLQEMLLGGSANEAPHAHHSLHPHLVQEHLSNNVIVTANMATPLHNGQ 384

zfHnf4α_tv5 FVKLFGMAKIDNLLQEMLLGGSANEAPHAHHSLHPHLVQEHLSNNVIVTANMATPLHNGQ 393

zfHnf4α_tv2 FVKLFGMAKIDNLLQEMLLGGSANEAPHAHHSLHPHLVQEHLSNNVIVTANMATPLHNGQ 387

zfHnf4α FVKLFGMAKIDNLLQEMLLGGSANEAPHAHHSLHPHLVQEHLSNNVIVTANMATPLHNGQ 406

zfHnf4α_tv7 FVKLFGMAKIDNLLQEMLLGGSANEAPHAHHSLHPHLVQEHLSNNVIVTANMATPLHNGQ 379

****************************:********************:*****:****

gcHnf4α MSTPETPIPSPPTASGSDHYKMAPGVIATVPKQPSSIPQPTITKQEAI 453

zfHnf4α_tv4 MSTPETPIPSPPTASGSDHYKMASGVIATVPKQPSSIPQPTITKQEAI 446

zfHnf4α_tv3 MSTPETPIPSPPTASGSDHYKMASGVIATVPKQPSSIPQPTITKQEAI 468

zfHnf4α_tv1 MSTPETPIPSPPTASGSDHYKMASGVIATVPKQPSSIPQPTITKQEAI 440

zfHnf4α_tv6 MSTPETPIPSPPTASGSDHYKMASGVIATVPKQPSSIPQPTITKQEAI 432

zfHnf4α_tv5 MSTPETPIPSPPTASGSDHYKMASGVIATVPKQPSSIPQPTITKQEAI 441

zfHnf4α_tv2 MSTPETPIPSPPTASGSDHYKMASGVIATVPKQPSSIPQPTITKQEAI 435

zfHnf4α MSTPETPIPSPPTASGSDHYKMASGVIATVPKQPSSIPQPTITKQEAI 454

zfHnf4α_tv7 MSTPETPIPSPPTASGSDHYKMASGVIATVPKQPSSIPQPTITKQEAI 427

*********************** ************************
